# Electrical Stimulation of Oral Tissue-Derived Stem Cells: Unlocking New Potential for Dental and Periodontal Regeneration

**DOI:** 10.3390/cells14110840

**Published:** 2025-06-04

**Authors:** Rúben S. Pires, Mafalda S. Santos, Filipe Miguel, Cláudia L. da Silva, João Carlos Silva

**Affiliations:** 1 Department of Bioengineering, iBB-Institute for Bioengineering and Biosciences, Instituto Superior Técnico, Universidade de Lisboa, Av. Rovisco Pais, 1049-001 Lisboa, Portugal; ruben.pires@tecnico.ulisboa.pt (R.S.P.); mafaldasantos4@tecnico.ulisboa.pt (M.S.S.); filipearmiguel@tecnico.ulisboa.pt (F.M.); claudia_lobato@tecnico.ulisboa.pt (C.L.d.S.); 2 Associate Laboratory i4HB—Institute for Health and Bioeconomy, Instituto Superior Técnico, Universidade de Lisboa, Av. Rovisco Pais, 1049-001 Lisboa, Portugal

**Keywords:** electrical stimulation, oral tissue-derived mesenchymal stem/stromal cells, dental/periodontal tissue engineering, osteogenic differentiation

## Abstract

The tooth and its supporting periodontium are essential structures of the oral cavity, frequently compromised by conditions such as dental defects, aries, and periodontal diseases, which, if poorly treated, often lead to tooth loss. These conditions, affecting billions of people worldwide, remain significant healthcare and socio-economic challenges. Regenerative dentistry has emerged as a possible therapeutic option, leveraging advances in tissue engineering (TE), stem cell biology, and biophysical stimulation. Oral tissue-derived mesenchymal stem/stromal cells (OMSCs) hold great potential for dental and periodontal regeneration. Electrical stimulation (ES), a biophysical cue known to regulate key cellular behaviors such as migration, proliferation, and differentiation, has gained increasing attention for enhancing the therapeutic capacities of OMSCs. This review explores the biological properties of OMSCs under ES, its role in regenerative dentistry, and recent breakthroughs in ES-based dental and periodontal TE strategies. Furthermore, the current challenges and future directions for translating these innovative approaches into clinical practice are discussed.

## 1. Introduction

The tooth is a complex and long-lived organ in which soft connective tissue, known as dental pulp, is encased within an inextensible chamber composed of mineralized hard tissues, including enamel, dentin, and cementum [1]. The tooth is supported by the periodontium, a complex structure made up of both hard and soft tissues: alveolar bone, root cementum, periodontal ligament (PDL), and the portion of the gingiva that faces the tooth (dentogingival junction/gingiva) [2]. The alveolar bone is the part of the maxilla or mandible that houses the sockets surrounding and anchoring the teeth. It is a hard tissue that is highly mineralized and composed of 60% (w/w) inorganic material, 25% (w/w) organic material, and 15% water [3]. In turn, the cementum is a hard, avascular connective tissue that surrounds the roots of teeth, responsible for attaching the main PDL fibers. The cementum composition is highly similar to that of the alveolar bone, being composed of 12% water, 23% (w/w) organic material, and 65% (w/w) inorganic material [2,3]. The PDL is a soft, complex connective tissue placed between the inner wall of the alveolar bone socket and the cementum, which covers the tooth [2,4]. The extracellular compartment of the PDL is composed of collagen fiber bundles that are highly aligned and structured, as well as of non-collagenous matrix components such as glycoproteins and proteoglycans [2,5,6]. The dentogingival junction, also referred to as the region of the gingiva interacting with the tooth, is an adaptation of the oral mucosa that includes epithelium and connective tissue elements [2].

The most critical oral health conditions include dental caries, periodontal diseases, tooth loss, and oral/pharyngeal cancers [7]. In 2015, the estimated number of people with untreated oral conditions reached 3.5 billion, a significant 40% increase from 1990. Of the 313 diseases analyzed in the 2015 Global Burden of Disease Study, untreated caries in permanent teeth were the most prevalent, affecting 2.5 billion people worldwide; untreated caries in deciduous teeth affected 513 million children; severe periodontal disease affected 538 million people; and 276 million people experienced total tooth loss [8,9]. The global economic burden of the five main oral conditions is reported to be approximately USD 710 billion, of which USD 387 billion were direct costs, and USD 323 billion were related to productivity losses. These five main conditions include caries of deciduous teeth, caries of permanent teeth, chronic periodontitis, edentulism (total tooth loss), and other disorders (a heterogeneous group including a variety of tooth, tongue, and jaw disorders and malformations not included in the other causes) [10]. Periodontal diseases encompass a range of conditions, from gingivitis to early, moderate, and severe periodontitis. These conditions affect the tooth supporting tissues, namely alveolar bone, root cementum, PDL, and gingiva, and are characterized in their advanced forms by the loss of the tooth attachment, which can lead to tooth loss [11]. In 2018, the economic burden of periodontal diseases alone was estimated to be USD 154.06 billion in the United States and EUR 158.64 billion in Europe [12].

Since the 1950s, numerous clinical techniques have been developed to improve dental and periodontal regeneration. These include surgical procedures, root surface conditioning, bone grafts, guided tissue regeneration (GTR) using barrier membranes, and the application of growth factors. Recent advances in stem cell research and tissue engineering (TE) have resulted in promising approaches to regenerative dentistry. The identification of stem/stomal cells in postnatal dental and periodontal tissues has offered new possibilities for cell-based and TE strategies for periodontal regeneration [13]. While better oral hygiene and supragingival scaling can reverse dental biofilm-induced gingivitis, which constitutes the initial stage of periodontal disease, more severe periodontal disease requires surgical intervention. Surgical therapies for periodontal disorders are occasionally combined with antimicrobial agents and systemically delivered antibiotics [14,15]. In the treatment of periodontal disease, scaling has been the most popular clinical method for physically removing the calculus and biofilms [16]. However, while scaling is helpful at controlling infection and inflammation, it cannot promote tissue regeneration or fully restore damaged periodontal structures. This limitation emphasizes the importance of advanced regenerative techniques, such as TE procedures, to aim for a more complete and functional periodontal regeneration.

TE is an interdisciplinary field that uses principles from cell and developmental biology and biomaterials science to create new engineered tissues to replace lost or damaged ones [17]. An adequate biomaterial construct combined with responsive target cells and specific regulatory signals (e.g., chemical or physical cues) are required for a successful TE strategy. Dental and periodontal TE approaches often involve utilizing progenitor cells and instructive signals within prefabricated three-dimensional (3D) constructs that are then transplanted into the lesion site [18]. These approaches address some of the limitations associated with traditional regenerative dentistry procedures, since directly delivering growth factors and target cells overcomes the normal lag phase of progenitor cell recruitment to the defect site [13]. Mesenchymal stem/stromal cells derived from sources such as the bone marrow, PDL, and dental pulp are of special importance in periodontal wound healing and repair, as they have the capacity to differentiate into osteoblasts, cementoblasts, and fibroblasts, responsible for the regeneration of damaged periodontal tissues [13,19,20,21]. A better understanding of these cells’ properties and regulation of their differentiation mechanisms is needed to enable their effective use for regenerative dentistry and periodontal therapy.

Endogenously generated bioelectric currents play a key role in important biological processes including embryogenesis, wound healing, tissue repair and remodeling, as well as the normal growth of organisms [22]. Both the cytoplasm and the extracellular space display endogenous electric fields (EFs), which range from a few to hundreds of mV/mm in intensity [23]. Electrical stimulation (ES) is a biophysical cue that can be applied to impact stem cell migration, proliferation, and osteogenic differentiation, all of which are critical to the success of bone TE therapies [24,25,26,27,28,29]. Therefore, ES has emerged as a promising strategy that could also be possibly explored for dental and periodontal TE.

To explore the potential synergy of ES and oral tissue-derived mesenchymal stem/stromal cells (OMSCs) for therapeutic use, a deeper understanding of the changes in their biological properties when exposed to this stimulus is necessary. Accordingly, this review explores the potential of OMSCs in regenerative medicine (RM), emphasizing the role of ES as a biophysical cue to influence their behavior. Recent advances in applying ES for dental and periodontal tissue regeneration are highlighted, and the current challenges and future directions in optimizing these approaches for clinical translation are also discussed.

## 2. Oral Tissue-Derived Mesenchymal Stem/Stromal Cells

**Figure 1:**
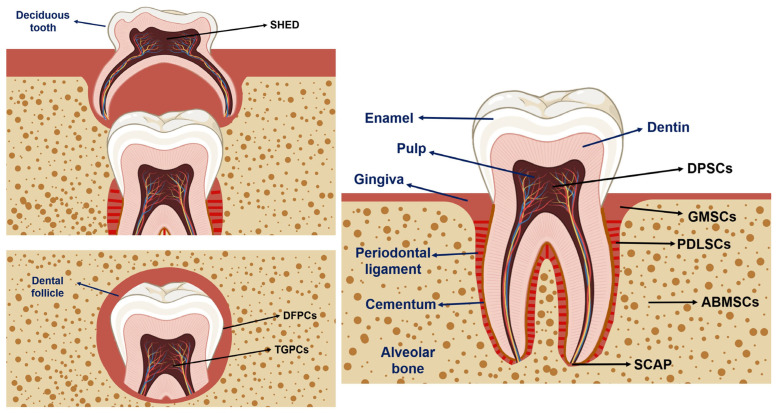
Schematic illustration of healthy adult and deciduous tooth (top left), depicting tooth anatomy and the main sources of oral tissue-derived stem/stromal cells. The adult tooth (right) is depicted with its major components, including the periodontium, composed of the PDL, alveolar bone, and cementum, as well as the enamel and dentin. Additionally, the dental follicle, a transient structure surrounding the developing tooth germ, is also represented (bottom left). Various populations of OMSCs can be isolated from different oral tissues, including alveolar bone-derived MSCs (ABMSCs), dental follicle stem/progenitor cells (DFPCs), dental pulp stem/stromal cells (DPSCs), gingiva-derived MSCs (GMSCs), periodontal ligament stem/stromal cells (PDLSCs), stem/stromal cells from the apical papilla (SCAP), stem/stromal cells from exfoliated deciduous teeth (SHED), and tooth germ stem/progenitor cells (TGPCs). Figure created using Biorender (https://www.biorender.com/, accessed on 8 Janurary 2025).

Since the 1970s, the search for mesenchymal stem/stromal cells (MSCs) in specific tissues has resulted in the discovery of unique populations of MSCs from a variety of human oral tissues. To date, eight distinct populations of OMSCs have been isolated and characterized (Figure 1): dental pulp stem/stromal cells (DPSCs), periodontal ligament stem/stromal cells (PDLSCs), stem/stromal cells from human exfoliated deciduous teeth (SHED), dental follicle stem/progenitor cells (DFPCs), alveolar bone-derived MSCs (ABMSCs), stem/stromal cells from apical papilla (SCAP), tooth germ stem/progenitor cells (TGPCs), and gingival MSCs (GMSCs). A chronological perspective on the discovery and identification of these eight OMSC populations is presented in the next paragraph.

In 2000, Gronthos et al. isolated DPSCs from adult humans and found that they could regenerate a dentin–pulp-like complex [30]. Three years later (2003), Miura and colleagues isolated a population of stem cells from the living pulp remnants of exfoliated deciduous teeth, which were named SHED. Human PDLSCs isolated from the PDL were described and characterized in 2004 by Seo et al. [31]. One year later, in 2005, Morsczeck and colleagues identified undifferentiated lineage-committed cells with mesenchymal progenitor characteristics in the dental follicles of impacted third molars, which were named DFPCs [32]. In the same year, MSCs were also discovered in the alveolar bone, namely ABMSCs [33]. Sonoyama et al. identified and isolated SCAP from the apical papilla tissue of an incompletely developed tooth in 2006 [34]. In 2008, Ikeda and colleagues reported a novel stem cell source, named TGPCs, which were isolated from discarded third molar, commonly referred to as wisdom teeth [35]. Finally, Zhang et al. published the first report on the isolation of a GMSC population in 2009 [36].

Besides their intrinsic capacity to self-renew and proliferate, these OMSCs have demonstrated strong tissue regenerative properties, in vitro multilineage differentiation potential, and advantageous immunomodulatory roles [37]. This differentiation capacity is typically characterized in vitro, where controlled environments allow for the assessment of lineage-specific markers and functional outcomes. Some of these characteristics are summarized in Table 1.

Bone regeneration is a crucial area in orthopedic and maxillofacial reconstruction, with stem cell-based therapies emerging as promising strategies to enhance healing and tissue repair. Among the various stem cell types, OMSCs have gained attention due to their ability to regenerate bone tissue in vivo [30,99]. Several studies have explored their regenerative potential, investigating different stem cell sources, scaffolds, and animal models to optimize outcomes. A study from Yamada et al. [100] investigated the use of stem cells from adult canines’ dental pulp and bone marrow and from puppies’ deciduous teeth to regenerate large bone defects, using platelet-rich plasma (PRP) as a scaffold. Following the creation of bone defects in the mandibles, several graft compositions were implanted, including PRP alone and combinations of bone marrow MSCs and DPSCs with PRP. The study demonstrated that stem cell-based grafts originated well-formed bone and neovascularization, with significantly enhanced bone regeneration when compared to the control and PRP-only groups, underlining the synergistic effect of PRP, DPSCs, and deciduous tooth stem cells in bone healing, envisaging applications in orthopedic and maxillofacial reconstruction. Building on this approach, further studies by the same group, using the same cell types in hydroxyapatite (HAp)-coated, osseointegrated dental implants, reinforced the previous findings, showing that HAp provided an improved microenvironment for stem cell adhesion and differentiation. This led to superior bone formation and better implant integration compared to control and PRP-only groups [100]. In a different model targeting periodontium reconstruction, Hu et al. [101] examined cell injection and cell sheet transplantation of DPSCs in swine periodontal bone defect models. The results demonstrated that both techniques were able to considerably replace alveolar bone; however, the cell sheet transplantation led to improved bone regeneration outcomes. This study suggests that not only the choice of stem/progenitor cell type, but also the method of delivery plays a critical role in optimizing bone TE strategies in the context of periodontal regeneration.

Several animal models, including rat, miniature pig, and beagle dog defect models, have been used to study the regenerative potential of PDLSCs in periodontal defects. The findings indicated that PDLSCs had the ability to create both soft and hard structures that resembled periodontal tissue, promoting its regeneration [102]. An approach introduced by Ding et al. [103], using allogeneic PDLSCs sheets applied to a miniature pig periodontitis model, showed significant periodontal tissue regeneration in both the autologous and allogeneic PDLSCs transplantation groups at 12 weeks post-PDLSCs transplantation, with no significant difference between the autologous and allogeneic groups. Furthermore, no immunological rejections were observed in animals receiving allogeneic PDLSCs transplantation. Notably, a different study from Chen and colleagues [104] assessed the safety and feasibility of employing autologous PDLSCs and grafting materials in a GTR strategy to treat periodontal intrabony defects. Patients were given either GTR with PDLSCs sheets and Bio-Oss^®^ or GTR with Bio-Oss^®^ alone. Over a 12-month follow-up period, no safety concerns of PDLSCs were detected, and both groups demonstrated significant alveolar bone regeneration. These findings offer preliminary clinical evidence for the effectiveness of OMSCs transplantation in regenerative dentistry.

## 3. Electrical Stimulation as a Relevant Tool to Modulate Cell Fate

Bioelectricity is crucial in coordinating cell activities during embryonic development, regeneration, and healing. It influences key processes such as cell proliferation, differentiation, alignment, and migration, all of which are essential for maintaining cell homeostasis, growth, and repair [105]. Bioelectrical circuits serve as long-range signaling mechanisms that regulate these important functions. Applying external electrical stimuli has been shown to enhance control over cell migration, orientation, growth and maturation [106,107,108]. Integrating bioelectricity into TE strategies could improve the quality and functionality of engineered tissues by providing a closer mimicry of the in vivo environment [108].

ES has drawn special interest since Fukada and Yasuda [109] described bone piezoelectricity in 1953, which has been correlated with the bones’ ability to adapt to mechanical stress and self-regenerate [110]. The advantageous therapeutic effects of ES observed in bone regeneration strategies in vivo motivated the TE and RM scientific community to further investigate the underlying cellular processes [111,112]. Additionally, the combination of ES treatment and osteogenic induction medium has been shown to upregulate important osteogenic-related markers both for bone marrow-derived MSCs and adipose tissue-derived MSCs [26,113,114,115].

ES has also been proven to modulate stem cells towards various fates beyond the osteogenic lineage. ES enhances fetal neural stem cell proliferation and differentiation into neurons [116], promotes guided cell migration and integration [117], as well as embryonic stem cell differentiation [118] towards the neurogenic lineage. ES has been applied to several cell types to promote cardiac differentiation, including human MSCs [119,120], human cardiac progenitor cells [121], mouse adipose tissue-derived MSCs [122], embryonic stem cells (both human and mouse) [23,123], and induced pluripotent stem/stromal cells [124]. Chondrogenic [125,126,127,128] and angiogenic [129,130,131] differentiation protocols using ES have also been reported. Altogether, ES has been proven to be an effective external biophysical stimulus to control the behavior of various types of stem cells, promoting their differentiation into specific lineages.

### 3.1. Piezoelectric/Electrical Properties of Dental and Periodontal Tissues

Understanding the piezoelectric and electrical properties of dental and periodontal tissues is critical for developing novel therapeutic strategies because it serves as the foundation for advances in regenerative dentistry and TE, as well as guiding future ES research and clinical applications.

Piezoelectricity is an intrinsic property of many biological tissues including bone, enamel, cementum, dentin, and PDL. This phenomenon occurs when the application of mechanical loads in the tissue results in the generation of electrical potential (and vice versa) [132,133]. Braden et al. [134] first reported that elephant dentin was piezoelectric, meaning that electric potentials arose from compressive stresses. In their study, the piezoelectric effect could not be seen with enamel. Marino and Gross [135] measured the piezoelectric constants of whale dentin and cementum and compared them to those of bone. In their study, dentin and cementum specimens were capable of producing (on average) around 12% of the surface charge density produced by cortical bone under comparable mechanical loading conditions.

The significance of piezoelectricity arises from its ability to promote tooth healing and growth in a similar way that it does for bone. The mineral and organic components in enamel and dentin give teeth their piezoelectric properties. The piezoelectricity of tooth HAp was evaluated in both dentin and enamel, and the results revealed that dentin had a higher piezoelectricity than enamel. It was discovered that piezoelectricity decreased in tooth samples after the organic material was removed, highlighting the importance of organic components in piezoelectric properties [136]. In fact, the enamel mineral components alone were reported to not be piezoelectric [137]. Moisture content and tubule orientation both influence dentins’ piezoelectric capabilities. When compressive stress was applied, greater voltages were created parallel to the tubules rather than vertically. The piezoelectric constant of dentin was shown to increase with moisture content and was classified as anisotropic: dry dentin piezoelectric constant was at 0.23 pC/N parallel to tubules and 0.075 pC/N vertically. At 1.93% moisture, values were measured as 1.16 pC/N and 0.87 pC/N. The highest values (4.56 pC/N and 4.09 pC/N) were obtained at 4.20% moisture. These findings highlighted dentin electromechanical behavior, which was found to be significantly influenced by hydration and structural anisotropy [138]. Different pH environments were designed to investigate the effect of pH on dentins’ piezoelectric properties. Researchers discovered no significant differences between the groups when the force was delivered vertically to the dentinal tubules. However, data showed that the alkaline group had stronger dentin piezoelectricity (1.347 pC/N) than the neutral (1.252 pC/N) and acidic (1.082 pC/N) groups. Significant changes were observed when the force was applied along the dentinal tubules. The alkaline group exhibited the highest piezoelectricity (6.079 pC/N), followed by the neutral (4.360 pC/N) and acidic groups (2.868 pC/N). These findings indicated that an alkaline pH environment increased dentin piezoelectricity, especially when force was applied along the tubules’ direction [132].

Teeth vary in morphology, so it is reasonable to predict that the surrounding tissues will have diverse electrical properties from subject to subject. In vitro measurements of the electrical properties of teeth showed that dentin has a conductance of 5 × 10^−3^ S/m and a relative permeability of 1 × 10^4^ at 1 kHz, and a conductance of 6 × 10^−3^ S/m and a relative permeability of 4 × 10^3^ at 10 kHz. At 1 kHz, the root canal has a conductance of 0.25 S/m and a relative permeability of 4 × 10^4^. At 10 kHz, the conductance remains 0.25 S/m and the relative permeability is 1 × 10^4^ [139]. Enamel is a non-electrically conductive component; at room temperature, it acts as an insulator. At approximately 25 °C, human tooth enamel has an impedance of around 1 × 10^15^ Ohms. The resistance of tooth enamel is high, yet it coexists with low resistance zones caused by hypermineralization. The human tooth enamel is highly resistive to electric conduction, but dentin exhibits a constant resistance regardless of position in the tooth [140].

The electrical properties of periodontal tissues are influenced by the tooth roots’ structure and surrounding tissues. The root canal system is surrounded by dentin and cementum, both of which serve as electrical insulators. At the minor apical foramen, a small conductive pathway connects the materials inside the canal (such as tissue and fluid) to the PDL, which is a conductor of electric current (EC). In this context, dentin, along with the tissue and fluid within the canal, forms a resistor whose value depends on the dimensions and inherent resistivity of these materials. In addition to resistive characteristics, the tooth root has capacitive properties, which create a dielectric constant defined by the fluid and tissue within the canal, as well as the cementum and dentin walls. This complex combination of resistive and capacitive components impacts the overall impedance properties of the root canal system and surrounding periodontal tissues [141].

The biomechanical behavior of periodontal tissues was investigated in both porcine and human models, offering insights into potential piezoelectric properties, even though they were not directly measured. In the porcine model, forces applied to the PDL reduced over time, indicating a viscoelastic response to varied loading velocities. Measurements in human samples, however, demonstrated a more fluid-dependent behavior in the PDL, with force parameters changing after each loading cycle. Understanding these behaviors may provide insights into the possible role of piezoelectric effects in periodontal tissue remodeling and healing [142]. Mechanical loading activates Piezo1 ion channels in human PDL cells, which leads to osteoclastogenesis. The nuclear factor (NF)-κB signaling pathway affects the expression of genes related to bone resorption, including *Cox2* and *RANKL*. Piezo1 ion channels might play a role in the mechanotransduction of mechanical forces in periodontal tissues, which influences alveolar bone remodeling [143].

A capacitive coupling (CCoupling) EF (sinusoidal wave, 60 kHz, and 5 V peak-to-peak) delivered to dogs, for 14 h per day, resulted in reorganization of the bony raspings on the electrically stimulated sides. These raspings are made up of particles from an asymmetrical crystal class that has piezoelectric properties, which means they generate electrical currents when exposed to mechanical pressures. The existence of these piezoelectric particles in adequate concentration is thought to have contributed to the observed alterations in bone structure, which were connected to bone remodeling or healing processes in response to ES [144]. Bioimpedance measures compared healthy periodontal tissue to inflammatory tissue. Simulations at 150 kHz revealed that healthy tissue had a higher impedance, but inflammatory tissue has lower values, indicating higher electrical conductivity. These observations suggest that bioimpedance may accurately distinguish between healthy and diseased tissues [145].

### 3.2. ES Delivery Methods

**Figure 2:**
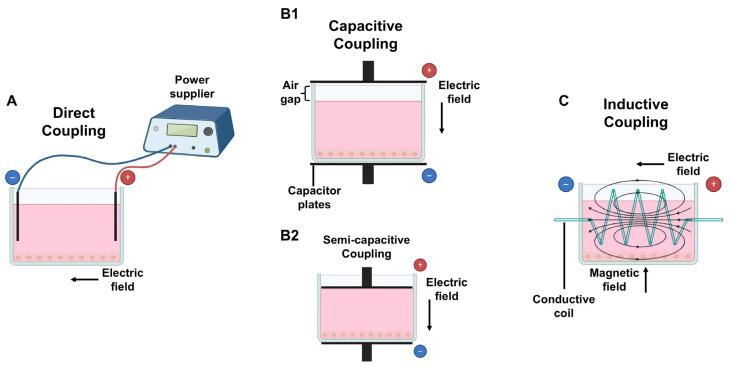
Methods to deliver electrical stimulation (ES) to cultured cells/TE constructs: (**A**) direct coupling: electrodes are placed inside the medium; (**B1**) capacitive coupling: capacitor plates are placed outsider the medium with an air gap on top; (**B2**) semi-capacitive coupling: similar to B1 without an air gap; (**C**) inductive coupling: a conductive coil surrounds the medium, creating both an electric and magnetic field, perpendicular to each other. Figure created using Biorender (https://www.biorender.com/, accessed on 8 Janurary 2025).

The main three methods to apply ES have been previously described and are depicted in Figure 2: direct coupling (DCoupling), CCoupling, and inductive coupling (ICoupling) [108,146,147].

The DCoupling approach (Figure 2A) involves placing conductive electrodes (such as graphite, platinum wires, or stainless steel) directly inside the cell culture wells or stimulation chamber, ensuring direct contact with the culture medium. This setup is commonly used in petri dishes or culture chambers for in vitro DCoupling experiments [148] and is the most widely used method because of its easy operation [149]. While inserting electrodes into the culture medium represents a simple procedure for ES treatment, it can also trigger undesirable side effects including redox reactions with medium components, electrode corrosion, and the formation of reactive by-products such as hydrogen peroxide, hydroxyl ions, and free radicals. Additionally, electrochemical reactions may alter pH levels or cause metal oxidation, leading to potential contamination of the cell culture medium [150,151]. These disadvantages could be overcome by electrodes insulation to deconstruct the impacts of DCoupling ES and Faradaic by-product generation on cellular activities [150].

Another DCoupling experimental setup for applying EFs to cells or tissues is to create an isolated chamber with agar salt bridges connecting it to exterior silver (Ag)/Ag-chlorine (Cl) electrodes immersed in Steinberg solution [149]. This design can separate EF-exposed cells from reactive Faradaic electrolysis products (hydrogen peroxide, hydroxyl and superoxide ions, or other free radical intermediates) produced by redox reactions at the electrode–electrolyte interface [150]. Accordingly, this is crucial, since the adsorption of proteins to the electrodes can disrupt the current, decreasing ES intensity and DCoupling efficacy [151].

CCoupling (Figure 2B1) is a non-invasive ES approach that involves creating EFs between two parallel conductive layers, known as capacitor plates, connected to a generator. These are located at the edges of the cell culture well or chamber, usually above and below the cell culture media, but not in contact with it, leaving a small air gap between the upper conductive layer and the cell culture medium in the well. If there is no space between the medium and the top capacitor plate, the method is referred to as semi-capacitive coupling (Figure 2B2) [111]. The EF generated is uniformly transmitted through the culture media, and the cells are evenly stimulated, regardless of their position in the culture well [152,153].

ICoupling (Figure 2C) stimulation involves generating EFs surrounding the cell culture system using a conductive coil or solenoid. An alternating current flows through the coil, originating a magnetic field and a perpendicular alternating EF [154], therefore creating an electromagnetic field (EMF). This stimulation approach prevents direct cell contact with the electrodes, which might eliminate the presence of unwanted by-products [111].

### 3.3. ES Parameters

There are several ES parameters that can be controlled to influence cell behavior. ES can be delivered in the form of EC, measured in amperes (A), or electrical potential differences, measured in volts (V). EFs exist whenever charge is present, and its strength is measured in volts per centimeter (V/cm), expressed as intensity for field strength. An EF of 1 V/cm represents a potential difference of 1 V between two points that are 1 cm apart. If the exposure time and EF strength are within tolerance limits, treating biological systems or cells with EFs can result in positive biochemical and physiological reactions [155]. Units of magnetic flux density (intensity) are measured in either Gauss (G) or Tesla (T) [156]. ES uses a field or current that can be either direct (DC) or alternating (AC). AC changes the direction of the field/current at a certain period “τ” or frequency (1/τ). Research on EFs generated through AC is less common but interesting, since the application of AC (rather than DC) is often utilized to prevent ion buildup near electrodes, which can reduce the applied field or current [157]. The use of intermittent or continuous stimulation is another important parameter. If intermittent, the frequency and duration of each ES application should also be considered.

ES can be provided in the form of monophasic (single phase: unidirectional pulse from baseline to either positive or negative phase) and biphasic (two phases: bidirectional wave with one positive phase and one negative phase) wave [158,159,160,161]. The waveform can have different shapes, such as rectangular pulse, sinusoidal, square, triangular (center or increasing/decreasing ramp), sawtooth, and exponential (increasing/decreasing) [162,163,164]. Duty cycle is defined as the percentage of time the waveform period is “*on*” or active, representing the duration during which the cells are being stimulated, compared to the total cycle time, which includes both the active (on-time) and recovery (off-time) periods. The duty cycle of the waveform can be calculated using the equation % duty cycle = (t_p_ × f) × 100, where t_p_ is the pulse width, and f is the frequency of the wave [165,166,167]. According to the stimulation parameters setup, monophasic stimulation is effective in polarizing the target tissue [168], but it may produce reactive oxygen species (ROS) through the oxidation–reduction process due to the Faradaic reaction at the surface of a metal electrode [169]. Additionally, the Joule heating effect may cause cell damage, particularly when massive current pulses are delivered over long periods of time and/or at high frequencies [170].

Among various parameters, EF intensity may determine the osteoinductive windows. A study using osteogenic differentiation medium and AC EFs (2 mV/mm, 60 kHz, 40 min/day) found that the EF could induce a delayed osteogenic differentiation of MSCs [127]. However, Hronik-Tupaj et al. reported that a 2 mV/mm, 60 kHz EF promotes osteogenesis [113]. The observed discrepancy could be explained by the utilization of osteogenic molecules, which could have a synergistic effect with EFs to promote osteogenesis more effectively. Another research study from Hess et al. [171] revealed that EFs (0.36 mV/mm, 10 Hz) alone fail to induce osteogenic differentiation, but, when an osteogenic sulfated hyaluronan derivative is introduced, MSCs osteogenesis increases significantly. Therefore, EFs with low intensity may not promote MSCs differentiation toward osteoblasts by itself but could enhance the osteoinductive properties of biochemical molecules. High intensity above 100 V/cm can cause cell membrane electroporation, or an immediate increase in intracellular calcium ions (Ca^2+^) and ROS, followed by cell apoptosis [172,173]. Different from low-intensity EFs, moderate to high-intensity EFs could promote the osteogenic differentiation of MSCs. For instance, bone marrow-derived MSCs exposed to an EF of 100 mV/mm (which can be considered a moderate intensity rather than high) for 1 h per day undergo osteogenic differentiation. These osteoinductive effects were maintained even after EF was terminated. However, it is important to note that the cells were cultured in a medium supplemented with osteogenic factors, which may have contributed to the observed differentiation [24]. Khaw et al. [174] applied two different EFs (100 and 200 mV/mm) to human bone marrow-derived MSCs without biochemical osteogenic supplements and found that both EF conditions could promote the osteogenic differentiation, with the 200 mV/mm EF being the best-performing one. Ravikumar et al. [175] designed an EF device able to apply a static potential (15 V) to parallel electrodes separated by 15 mm. MSCs were seeded on HAp-CaTiO3 composites and then exposed to EFs for 10 min per day in the absence of osteogenic inductive molecules. The results demonstrated that the EFs could significantly improve the expression of osteogenic gene markers alkaline phosphatase (*ALP*), collagen type I (*COL I*), and osteocalcin (*OC*). These findings suggest that an EF with an intensity over 100 mV/mm may be required to promote MSCs osteogenesis in the absence of osteogenic molecules supplementation. Additionally, Silva et al. [114] verified variations of cellular responses, mainly regarding osteogenic differentiation, when human bone marrow-derived MSCs were subjected to five different ES protocols. Different ES protocols were applied every two days from day 0 to day 14. Conditions included basal and osteogenic medium without ES, as well as various ES settings: a constant DC EF of 1.2 V (for either 1 h or 1 s), a constant DC EC of 0.03 mA (for 1 h), and an AC EF of 1.2 V with ON/OFF periods of either 10 or 2 s (for 1 h). Results demonstrate that the current-controlled protocol effectively promoted human bone marrow-derived MSCs osteogenic differentiation, evidenced by enhanced in vitro mineralization and increased *OPN* gene expression.

Regarding the frequency, EMF frequencies below 1 kHz influence the cell cycle by decreasing the proportion of cells in the G1 phase while increasing those in the S phase. This shift enhances proliferative potential, as more cells undergo DNA synthesis, promoting overall cell proliferation and growth [176]. On the other hand, experiments suggest that ES frequencies above 1 kHz can induce cell differentiation, but must maintain low-intensity [125,127]. Numerous biological processes are impacted by very low frequency (0–100 Hz) EMFs, such as cell fate and differentiation, gene expression, and the release of essential growth factors [156].

Altogether, these findings demonstrate that a unique protocol for ES on MSCs has not been defined yet and should be pursued to perform more complex and comprehensive studies envisaging future clinical translation.

### 3.4. Cellular Mechanisms Affected by ES

Cells are the primary source of bioelectricity in living organisms [22]. A membrane potential, or V_mem_, is created when ions are continuously pumped through ion channels, causing a voltage gradient across their membrane [108]. The types of stem cells and the treatments’ goal determine the ES parameters (frequency, duration, voltage, and field/current) to use [177]. ES exposure can cause biophysical changes at the cell surface, altering its permeability, and affecting membrane protein functions such as enzyme activity (sodium (Na^+^)/potassium (K^+^) adenosine triphosphatase (ATPase) and Ca^2+^ ATPase), membrane–receptor complexes, and ion-transporting channels by changing the charge distribution of these biomolecules at the surface of the cells [146,178]. Specifically, voltage-gated ion channels react to variations in V_mem_ by opening or closing [156].

The complex interactions between cells and their chemical/physical environment are known to be affected by ES, which is crucial for preserving the regular biological processes of the body [179]. Studies have demonstrated that ES modulates intracellular calcium levels via calcium/calmodulin (CaM) pathways [108,180], which are important in the production of intracellular molecules, such as CaM-dependent kinases and phosphatases [181]. Additionally, further research has demonstrated the involvement of growth factor receptors in ES transduction, such as those for fibroblast growth factor (FGF), epidermal growth factor (EGF), transforming growth factor-beta 1 (TGF-β1), and vascular endothelial growth factor (VEGF) [182,183,184]. Moreover, ES promotes growth factor production by cells, such as bone morphogenetic proteins (BMPs: e.g., BMP2 and BMP4), TGF-β1, and insulin-like growth factor 2 (IGF-2) [179,185,186].

The influence of the inflammatory microenvironment on stem cell behavior is particularly relevant in periodontal regeneration. OMSCs are known to respond dynamically to inflammation: under high inflammatory conditions, they suppress inflammation and bone resorption by regulating cytokine secretion, immune cells activity, and RANKL/OPG signaling. Conversely, under low inflammatory conditions, they may promote mild inflammation and osteoclastogenesis as part of tissue remodeling [187]. Liu et al. [188] demonstrated that PDLSCs derived from inflamed periodontal tissues exhibit enhanced proliferation and migration but reduced osteogenic differentiation. This was attributed to elevated canonical wingless-related integration site (Wnt)/β-catenin signaling, which suppressed the noncanonical Wnt/Ca^2+^ pathway that supports osteogenesis. Pharmacological inhibition of β-catenin using dickkopf-related protein 1 (DKK-1) restored noncanonical signaling and rescued the osteogenic potential of these cells, highlighting the critical role of Wnt pathway balance in bone regeneration under inflammatory conditions.

In this context, ES has emerged as a promising biophysical strategy to modulate Wnt signaling and restore osteogenic capacity in OMSCs affected by chronic inflammation. In vitro studies have shown that ES can directly influence Wnt pathway activity. For example, human DPSCs exposed to pulsed EMF (PEMF) at an intensity of 10 mT for 15 min per day at varying frequencies (40, 60, 70, and 150 Hz) exhibited frequency-dependent effects on differentiation. The most pronounced osteogenic responses occurred at 60 and 70 Hz, with increased expression of β-catenin and phosphorylated GSK-3β, indicating activation of canonical Wnt signaling and promotion of odontoblast-like differentiation [189]. A complementary approach by Zhang et al. [190] further highlighted the therapeutic potential of bioelectrical environments for restoring osteogenesis in oral inflammatory conditions. The researchers engineered poly(vinylidene fluoridetrifluoroethylene [P(VDF-TrFE)] membranes with controlled surface potentials to mimic endogenous osteogenic membrane potentials (–55 mV), rather than applying conventional EC. When DPSCs were cultured on these surfaces, fibronectin adsorption was enhanced, leading to canonical Wnt activation and osteogenic differentiation. This study demonstrates that modulating the electrical characteristics of the cellular microenvironment can effectively direct OMSCs towards Wnt-mediated osteogenic commitment. Such approaches offer promising avenues for reestablishing bone-forming capacity in diseases where chronic inflammation disrupts regenerative signaling.

PEMF stimulation has also been shown to promote osteogenic differentiation in adipose tissue-derived MSCs through activation of canonical Wnt signaling. In vitro exposure to a 50 Hz, 20 mT PEMF, combined with zinc sulfate supplementation, led to significant upregulation of *Wnt1*, *Wnt3a*, *LRP5*, and *β-catenin*, along with increased expression of osteogenic markers. These findings provide further evidence that ES can enhance osteogenesis in MSCs by engaging the Wnt/β-catenin pathway under defined stimulatory conditions [191]. Moreover, PEMF has demonstrated the ability to promote osteogenic differentiation even under inflammatory conditions. In a study using adipose tissue-derived MSCs exposed to TNF-α, PEMF stimulation restored osteogenic potential through activation of the mTOR signaling pathway, a central regulator of stem cell fate and differentiation. These findings provide additional support for the role of ES in counteracting inflammation-induced suppression of osteogenesis, complementing its Wnt-mediated effects [192].

By modulating both canonical and noncanonical Wnt signaling, and potentially additional pathways, ES represents a multifaceted strategy to restore bone-forming capacity in inflamed oral environments, which may complement pharmacological Wnt-targeted therapies.

**Figure 3:**
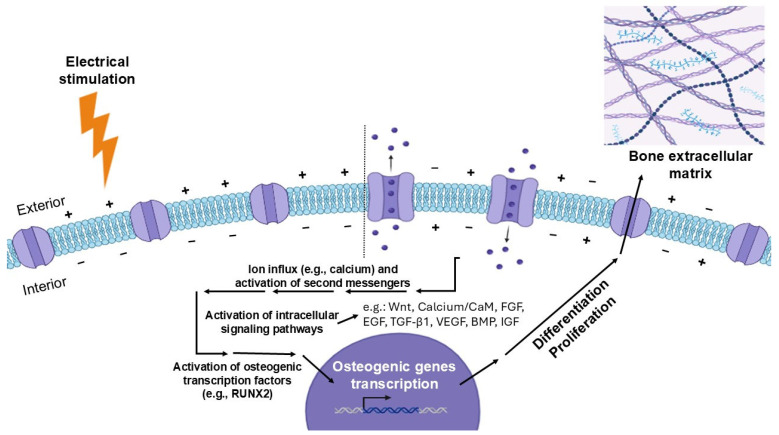
Visual representation of the biological processes occurring outside and inside a stem/stromal cell after electrical stimulation treatment in vitro. The membrane potential becomes altered, passing from a resting potential state (left side from the dots bar) to a hyperpolarized state (right side from the dots bar). A chain of cellular and molecular events starts to activate osteogenic gene transcription at the nucleus. Stem/stromal cells activate mechanisms of proliferation and differentiation, leading to the deposition of bone ECM components and consequently bone tissue formation. BMP: bone morphogenetic protein; CaM: calmodulin; EGF: epidermal growth factor; FGF: fibroblast growth factor; IGF: insulin-like growth factor; RUNX2: runt-related transcription factor 2; TGF-β1: transforming growth factor-beta 1; VEGF: vascular endothelial growth factor. Wnt: wingless-related integration site. Figure created using Biorender (https://www.biorender.com/, accessed on 8 Janurary 2025).

According to these findings, electrical cues can modulate stem cell behavior, particularly in electroactive tissues (e.g., cardiac, neural, muscle, bone). Therefore, it has been demonstrated that ES can be an effective tool to regulate stem cell behavior, improving processes such as cell migration, proliferation, differentiation, and extracellular matrix (ECM) synthesis [193]. Figure 3 provides a representation of the effects ES might have on electroactive stem/stromal cells at the cellular level, from alterations in V_mem_ to the formation of new bone ECM resultant from the activation of osteogenic pathways.

## 4. Electrical Stimulation for Dental and Periodontal Tissue Regeneration Strategies

ES has emerged as a promising approach for enhancing the regenerative potential of MSCs in dental and periodontal TE applications. Nevertheless, ES of OMSCs remains largely underexplored. Table 2 summarizes examples of studies using different ES protocols on OMSCs to promote their osteogenic differentiation in two-dimensional (2D) cultures, focusing on dental and periodontal tissue regeneration strategies.

Different voltages of DC EFs were administered to rat-derived DPSCs, 1 h per day for 7 days. The authors discovered that, although 10 and 50 mV/mm of ES had no effect on metabolic activity or cell numbers, 100 mV/mm significantly reduced cell numbers, and 150 mV/mm caused cell death. At day 7, ES treatment enhanced gene expression of osteo/odontogenic markers such as *OC*, *RUNX2*, bone sialoprotein (*BSP*), and dentin matrix protein 1 (*DMP1*). However, 50 mV/mm of ES lowered collagen deposition and ALP activity at both time points (Days 7 and 14), whereas calcium deposition decreased at day 14 [194]. DPSCs cultured on tissue culture dishes within a micro-current ES (mES) system exhibited significantly enhanced proliferation at a working mES of 38 μA compared to the other conditions. This stimulation altered the expression of intracellular and extracellular proteins, thereby enhancing proliferation and significantly promoting osteogenesis through the upregulation of *OC* expression relative to non-stimulated cells [193]. Despite the promising potential of various OMSC types such as PDLSCs, GMSCs, SCAP, SHED, AB-MSCs, DFPCs, and TGPCs, no studies have yet explored their response to ES with DCoupling or CCoupling. This represents a significant gap in research, as these stem/stromal cell populations hold immense potential for improving dental/periodontal regenerative therapies and enhancing patients’ quality of life. Given the demonstrated benefits of ES in promoting cell proliferation, differentiation, and tissue regeneration in other stem/stromal cell types, future research should focus on exploring ES impact on more types of OMSCs to fully assess if it could be a potential therapeutic approach to treat dental/periodontal diseases.

ES with the ICoupling method that creates an EMF in the cell culture has been explored to a higher extent with OMSCs. Human DPSCs were cultured in odontoblast differentiation medium with dexamethasone, BMP2, TGF-β1, and FGF-2. Human DPSCs were then subjected to 10 mT PEMF at 40, 60, 70, and 150 Hz, for 15 min per day. Protein and gene expression varied in human DPSCs based on the frequency of the stimulation protocol. At 60 and 70 Hz, the expression of typical immunophenotypic markers for MSCs (CD73, CD105, CD146) decreased more than at other frequencies, but the expression of odontoblast-related markers such as β-catenin, phosphorilated-GSK-3β, and phosphorilated-p38 increased. Exposure to a PEMF intensity of 10 mT at 70 Hz had a significant effect on human DPSCs differentiation by increasing DMP1 and dentin sialophosphoprotein (DSPP) proteins expression, as well as *ALP* and *DMP1* osteo/odontogenic gene expression [189]. The impact of low frequency EMF on human DPSCs proliferation rate was studied for 7 days at intensities of 0.5 and 1 mT, with a frequency of 50 Hz and exposure time of 20 and 40 min of stimulation per day, respectively. MTT assay results showed that the intensity of 1 mT for 20 min per day had a higher effect on human DPSCs proliferation. All exposure groups had significantly higher survival and proliferation rates than the control group (with the exception of the 40 min, 1 mT group), as evidenced by MTT and DAPI staining assays [195]. After 20 min of 0.5 mT exposure, the survival intensity is much higher than in the other groups. Therefore, according to the obtained results, low frequency EMF promotes the survival of cells except for one case (40 min, 1 mT), even though the effective underlying mechanisms in this process are yet unknown. Furthermore, other authors demonstrated that PEMF-stimulated groups increased the expression of the osteo/odontogenic-related gene *DMP1*, compared to non-stimulated control groups [196]. Wang and colleagues evaluated the proliferation and osteogenic differentiation of human PDLSCs when exposed to PEMF (15 Hz, 1 h daily, varying intensities), transfected with BMP9, or subjected to both stimuli [197]. PEMF at various intensities had no influence on the proliferation of human PDLSCs and did not increase the proliferative capacity of BMP9-transfected cells. The combination of PEMFs (1.8 or 2.4 mT) and BMP9 stimulation had a synergistic effect on early and intermediate osteogenic genes, as well as protein expressions of RUNX2, ALP, and osteopontin (OPN), according to the respective quantitative reverse transcription-polymerase chain reaction (qRT-PCR) and Western blotting analyses. A more mature, mineralized ECM formation was also confirmed in BM9-transfected human PDLSCs through calcium deposits visualized by alizarin red S staining. In human PDLSCs, another research group assessed the effects of intermittent low frequency EMF exposure (6 h per day) for the standard differentiation period (28 days) and for 10 days with or without osteogenic differentiation medium. After 10 days of exposure, compared to controls, there was an increase in cell proliferation rate and de novo calcium deposition, and qRT-PCR and Western blot results revealed that collagen type I alfa 1 (*COL1A1*) and *RUNX2* gene expression and COL1A1, RUNX2, and OPN protein expression were upregulated [198]. Additionally, in a study developed by Lim et al. [199], human ABMSCs exposed to low frequency-pulsed EMF had a 15% increase in proliferation compared to untreated cells on day 5. Furthermore, within two weeks of exposure to low frequency PEMF, ALP activity increased significantly during the early stages of osteogenesis, as did mineralization near the midpoint. Low frequency PEMF also increased vinculin, vimentin, and CaM expressions in comparison to the non-stimulated control. CaM demonstrated that low frequency PEMF increased the expression of the osteogenesis-related gene *OC*.

**Table 2:** Examples of studies using different ES protocols on OMSCs to promote osteogenic differentiation.

Cell Source	Method to Deliver ES	ES Parameters	Main Findings	Reference
ABMSC	EMF	10 min/day for 4 and 14 days; 6 Gauss; 10, 50, 100 Hz.	Proliferation increased for EMF groups at 50 and 100 Hz (Day 5); ALP and mineralization enhanced in EMF group at 50 and 100 Hz (Day 14); vinculin, vimentin, and CaM expression increased for EMF groups; *OC* expression upregulated for EMF groups.	[199]
DPSC	DC EF	1 h/day for 7 days; 10, 50, 100, 150 mV/mm.	Metabolic activity decreased for 100 and 150 mV/mm; bone-specific gene expression increased: *OC*, *RUNX2*, *BSP*, and *DMP1*; collagen deposition decreased at days 7 and 14; ALP activity decreased at days 7 and 14; calcium deposition decreased at day 14.	[194]
DPSC	mES	2 h/day at an intermittent regimen (stimulation time: 38.62 s and rest time: 110.46 s) for 3 days; mES of 0.5, 38 and 75.5 µA.	Proliferation and *OC* expression increased for 38 µA stimulation condition.	[193]
DPSC	EMF	15 min/day for 3 days; 10 mT; 40, 60, 70, and 150 Hz; pulse waveform.	CD73, CD105, CD146 expression decreased for 60 and 70 Hz; RUNX2, DMP1, DSPP protein expression increased for 70 Hz; ALP, RUNX2, DMP1, and DSPP protein expression increased for 60 Hz; *BMP2*, *ALP*, *RUNX2*, *OMD*, *DMP1* and *DSPP* gene expression increased for all PEMF groups; β-catenin, p-GSK-3β, and p-p38 expression increased for 70 Hz; β-catenin expression increased for 60 Hz.	[189]
DPSC	EMF	20 and 40 min/day for 7 days; 0.5 and 1 mT; 50 Hz; sinusoidal waveform.	Proliferation increased for EMF groups, especially for 1 mT, 20 min/day.	[195]
DPSC	EMF	20 and 40 min/day for 7 days; 0.5 and 1 mT; 50 Hz; sinusoidal waveform.	Proliferation and *DMP1* expression increased for EMF groups.	[196]
DPSC	DC EF on PPy films	4 h/day for 2 and 4 days; 0.33 V/cm.	Calcium deposition, Alizarin red S staining, and *BMP2*, *BMP3*, *BMP4*, and *BMP5* gene expressions enhanced for ES group.	[200]
DPSC	Indirect ES on poralized P(VDF-TrFE) membranes	Membranes: P-55 (−55.05 mV), P-85 (−84.8 mV), and P-0 (unpolarized).	ALP activity increased for P-55 (day 7); mineralization increased for P-55 (day 21); BMP2, COL I, and SP7/OSX protein expression increased for P-55 (day 7); BMP2, COL I, ALP, SP7/OSX, OPN, VINCULIN, and OC protein expression increased for P-55 (day 14).	[190]
PDLSC	EMF	1 h/day for 4, 7, 8, 10, and 14 days; 0.6, 1.2, 1.8, 2.4, and 3.0 mT; 15 Hz; pulsed waveform.	Alizarin red S staining, ALP activity, *RUNX2*, *ALP*, and *OPN* gene expression increased for 1.8 and 2.4 mT; BMP9-transfected cells with PEMF increased even more these genes’ expression and ECM mineralization.	[197]
PDLSC	EMF	1 mT; 6 h/day; 10 or 28 days; 50 Hz; sinusoidal waveform.	Proliferation and calcium deposition, *COL I*, *RUNX2* gene expression, and COL I, RUNX2, OPN protein expression increased for 10-day treatment group.	[198]

ABMSC: alveolar bone-derived mesenchymal stem/stromal cell; ALP: alkaline phosphatase; BMP: bone morphogenetic protein; BSP: bone sialoprotein; CaM: calmodulin; CD: cluster of differentiation; COL I: collagen type I; DC: direct current; DMP1: dentin matrix protein 1; DPSC: dental pulp-derived stem/stromal cell; DSPP: dentin sialophosphoprotein; ECM: extracellular matrix; EF: electric field; EMF: electromagnetic field; ES: electrical stimulation; mES: micro-current electrical stimulation; OC: osteocalcin; OMD: osteomodulin; OPN: osteopontin; PDLSC: periodontal ligament stem/stromal cells; PEMF: pulsed EMF; PPy: polypyrolle; P(VDF-TrFE): poly(vinylidene fluoridetrifluoroethylene; p-GSK-3β: phosphorylated glycogen synthase kinase-3 beta; RUNX2: runt-related transcription factor 2; SP7/OSX: SP7 transcription factor/osterix.

Even though ES in simple 2D culture plate environments can be easily controlled, conductive films and scaffolds provide more consistent and uniform stimulation, enhancing the effectiveness of the ES signal. Additionally, they provide a closer mimicry of the ECM, offering a more supportive and biologically relevant substrate for osteogenic differentiation [201,202]. ES studies using conductive films have also been explored with OMSCs and are included in Table 2. In contrast, to the best of our knowledge, there are no studies combining three-dimensional (3D) conductive scaffolds, ES, and OMSCs.

A conductive polypyrrole (PPy) film-based ES device delivering DC EF was fabricated to investigate its effects on the osteogenic differentiation of human DPSCs. Results showed that applying a 0.33 V/cm EF for 4 h on days 0, 2, and 4 to cells cultured under osteogenic induction medium significantly enhanced calcium deposition nearly threefold, accelerated mineralization (as evidenced by alizarin red S staining), and upregulated mRNA expression of *BMP2*, *BMP3*, *BMP4*, and *BMP5* during early osteogenic differentiation. While the precise mechanisms remain unclear, the findings suggest that ES may influence osteogenesis through BMP-related signaling pathways, particularly via the upregulation of *BMP2*, which is known to modulate *RUNX2* expression. Additionally, the observed increase in *BMP3* expression raises the possibility of its involvement in modulating BMP signaling to enhance mineralization. ES combined with DPSCs-seeded PPy films revealed the potential of DC EF treatment in bone TE [200].

A study investigating the effects of polarized P(VDF-TrFE) membranes on the osteogenic differentiation of human DPSCs highlights the potential of indirect ES in TE. The membranes were polarized to create surfaces with distinct potentials: P-55 (−55.05 mV), P-85 (−84.8 mV), and P-0 (unpolarized). When human DPSCs were cultured on these membranes, P-55 exhibited significantly higher ALP production after 7 days and promoted more pronounced mineralization nodules after 21 days, when compared to the other groups. Protein expression analysis revealed that P-55 enhanced the expression of key osteogenic markers, including BMP2, collagen type I (COL I), and SP7 transcription factor/osterix (SP7/OSX) on day 7, as well as BMP2, COL I, ALP, SP7/OSX, OPN, VINCULIN, and OC on day 14 [190]. These findings highlight the role of electrically polarized substrates in modulating stem cell behavior, providing an innovative strategy for bone, periodontal, and dental TE strategies.

## 5. Challenges and Future Trends

Despite significant advances, dental/periodontal TE still faces several challenges, both scientific and non-scientific, that hinder its clinical translation. Limited understanding of the structural and functional properties of oral tissues remains a critical challenge in dental/periodontal TE. While biomimetic TE approaches are being developed with the aim of replicating the natural biological processes that occur in vivo, further research is needed to refine these strategies and enhance their effectiveness for clinical applications. These challenges emphasize the need for clear regulatory guidelines [203] to guarantee ethically sound and standardized research.

To ensure the successful clinical translation of ES strategies, various challenges must be addressed, including the complexity of administering ES therapy in the oral cavity, the need for clinical visits for ES administration, and the effective removal of dead bacteria. A more in-depth analysis of how ES therapy affects the diverse microbial species within the biofilm system is required. Furthermore, the daily administration of ES may present practical challenges for both patients and healthcare providers [204].

Regardless of the abovementioned challenges, the use of ES in dental/periodontal TE applications is gaining visibility among researchers and clinicians alike. As discussed previously, ES comprises a wide range of electrical parameters that can vary significantly between protocols. The fundamental constraint is that ES on OMSCs is currently underexplored, and a lack of consensus on the application methods and parameters of ES may contribute to conflicting research findings. It is difficult to determine ideal procedures and standardized protocols since the ES parameters utilized in existing studies are too diverse to be relied on by clinicians [205]. An important challenge to be addressed is the establishment of an optimized ES protocol that could be beneficial for the majority of these different OMSC types, leaving time to investigate further cellular/molecular mechanisms and signaling pathways involved in OMSCs response to ES, all aspects that are still poorly understood. The basic pathways that mediate EFs interactions with cells are frequently not fully understood mainly because of the arbitrary selection of EF parameters. Therefore, a detailed optimization of ES parameters, including field strength, duration, and frequency, would be necessary for the proper interpretation of the studies. This would allow the cell to bypass its initial, passive state and reach the threshold level for active response [206,207].

Exposing monolayer 2D cultures, 3D cultures, and tissue engineered constructs to EF is usually challenging, specifically for long-term culture experiments. In certain circumstances, such long-term experiments necessitate complex setups with large circuits and multiple wires coming in and out of incubators [146], which could increase the complexity of the experimental design and its maintenance. These concerns were addressed by proposing a miniature stimulation device that uses circuit layouts on tiny electrical boards and 3D-printed cell culture chambers with biocompatible cell arrays and on-board integrated circuits to investigate how various stimulation parameters affect cell behavior [147]. It is also worth noting the drawbacks of utilizing electrical circuits with fixed resistances/impedances, as the inherent impedance of cells and culture medium can change as a result of EF-induced cellular responses. Instead, such experiments necessitate variable voltage or current outputs that can account for changes in biological reactions [146].

Future research should focus on several key areas to enhance the clinical applicability of ES in dental and periodontal regeneration. A critical aspect to advance this field is the comprehensive in vitro characterization of OMSCs. There is still a gap in our understanding of the various OMSC populations and their specific responses to ES. It is crucial to thoroughly characterize these cells to assess their potential for tissue differentiation and maturation, particularly in the context of dental and periodontal regeneration and when exposed to ES. This characterization will help define how ES can modulate cellular behavior and improve regenerative outcomes.

To optimize the application of ES, computational models should be developed to predict and estimate the distribution and intensity of EFs within both cell culture wells and ES bioreactor devices. These in silico models will help improving experimental protocols, enabling more precise and effective use of ES to promote tissue regeneration. In parallel, the development of high-throughput ES devices could facilitate the rapid testing of various ES conditions, helping to optimize parameters for different types of OMSCs and tissues. Most mechanistic studies focus on a single ion or channel. However, even at the cell level, hundreds of ion channels interact to determine the bioelectric state. Similarly, the bioelectric state of tissues is the result of interactions between different cell types. High-throughput electrophysiology techniques could help us better understand the key cellular regulation mechanisms [205].

Exploring alternative, non-direct ES approaches, particularly ICoupling using EMFs without direct implant contact, provides a promising area for future research [204]. A new strategy is to use triboelectric nanogenerators (TENGs), to transform mechanical energy from bodily movements into electrical energy, potentially functioning as in situ therapeutic triggers [208].

Another promising route is the combination of OMSCs with electroactive materials, which can be either electroconductive or piezoelectric materials, to enhance the efficacy of ES. These materials can generate or conduct electrical signals, thereby providing more controlled and localized ES that could improve the differentiation of OMSCs. The development of biocompatible and wearable nanomaterials is essential for the future, as induced bioelectric changes are an essential category of effects to consider for bioengineers working on dental biomaterials aiming to direct stem cell differentiation and promote tissue regeneration [205]. A wireless piezoelectric hydrogel was developed to produce electrical signals under mechanical stress. Piezoelectric stimulation improved the osteogenic differentiation of inflammatory PDLSCs by increasing adenosine triphosphate (ATP) production. It also reconfigured an anti-inflammatory and pro-regenerative niche by altering macrophage phenotypes. This combination of bioenergetic activation and immunological regulation promotes in situ regeneration in periodontal inflammatory defects in rats [209]. An injectable piezoelectric hydrogel (PiezoGEL) was developed to produce electrical charges when stimulated by biomechanical vibrations, such as mastication and movement. In vitro, PiezoGEL significantly reduced pathogenic biofilm biomass, metabolic activity, and viable cell numbers, and its antibacterial effects were associated with decreased cell adhesion and increased oxidative stress. PiezoGEL also improved bone marrow-derived MSCs viability and osteogenic differentiation. It also successfully decreased periodontal inflammation and stimulated bone tissue regeneration in an in vivo mouse model [210].

The combined use of multi-physical stimuli, such as electrical and mechanical forces, could also be a key focus to advance periodontal/dental TE strategies. By mimicking the complex and dynamic environment of the periodontium and teeth, which are constantly subjected to mastication forces or external factors, this approach could stimulate enhanced tissue regeneration and more accurately replicate the physiological conditions under which OMSCs would naturally develop. An mEC (10 μA) applied on the tooth surface of cats combined with a mechanical force (60 g orthodontic force) enhanced bone deposition, indicating that these multi-physical stimuli could accelerate alveolar bone remodeling and orthodontic tooth movement [211]. Additionally, ES holds the potential to enhance angiogenesis, which could promote the formation of blood vessels in periodontal tissues and the pulp of the tooth. Vascularization is essential for the survival of regenerated tissues, and ES could also help support the growth and repair of nerve fibers, crucial for restoring sensory functions after dental and periodontal injury. In fact, applying 150 mV/mm EFs to a co-culture of human DPSCs and human umbilical vein endothelial cells (HUVECs) in 3D Matrigel significantly enhanced angiogenesis and promoted odontoblast differentiation. EF-induced pre-vascularized engineered dental pulp tissue not only had odontoblasts, but also developed a rich vascular network, with smooth muscle-like cells appearing around the blood vessels, contributing to blood vessel maturation and stability [212].

Biochemical stimuli should also be considered alongside physical stimulation to create a more physiologically relevant environment for OMSCs. The clinical application of EC could involve approaches, such as electrochemical therapy, which is sometimes coupled with antibacterial drugs to increase effectiveness [203]. The periodontium and the tooth are environments rich in signaling molecules, cytokines, and growth factors that regulate tissue formation, maintenance, and regeneration. Combining biochemical and biophysical stimuli could significantly enhance the regenerative potential of OMSCs. Research into how ES interacts with these biochemical signals will further elucidate the mechanisms driving effective tissue regeneration. Therefore, understanding how ES affects cellular signaling pathways involved in OMSCs differentiation will provide valuable insights into how to use this technology for regenerative dentistry applications [203].

Finally, long-term in vivo preclinical studies will be essential to evaluate and validate the safety, efficacy and integration of ES-treated OMSCs into dental and periodontal tissues. In fact, ES with a low-intensity mEC (10 μA/5 min) stimulated tissue responses in rats’ PDLs, reducing the number of granulocytes and increasing the number of fibroblasts, blood vessels, and osteoclasts. It also regulated the expression of growth factors TGF-β1, VEFG, and FGF, which are known for their role in cell/tissue growth and regeneration [213]. A significant focus should be placed on improving therapy duration through different application intervals and measuring their efficacy in addressing important integration, inflammation, and infection concerns. Comprehensive in vivo investigations using both infectious and non-infectious models are required to fully validate and extend the current findings [205]. Additionally, the development of personalized ES protocols designed for individual patients will be critical for maximizing treatment outcomes [204]. To achieve these goals, interdisciplinary collaboration between researchers (biologists and bioengineers) and clinicians will be crucial, ensuring that the full potential of ES in dental/periodontal TE is explored. Through these efforts, ES may soon become an effective therapeutic tool to facilitate dental and periodontal regeneration.

## 6. Conclusions

Dental and periodontal diseases are critical public health concerns with global prevalence, leading to both functional and aesthetic impairments that affect self-esteem. Moreover, these conditions impose a substantial socioeconomic burden through elevated treatment costs and productivity losses.

Dental and periodontal tissues exhibit intrinsic electromechanical properties, which play a critical role in their biomechanical behavior, biological signaling, remodeling, and healing. The piezoelectric nature of dentin, cementum, and PDL, together with variations in electrical conductivity and impedance, suggest the potential role of ES on the regeneration of these tissues.

ES has emerged as a promising tool to enhance OMSCs-mediated regeneration by promoting proliferation and directing osteo/odontogenic differentiation pathways. DC and AC EFs, and PEMFs have shown promising results in OMSCs studies, significantly impacting cell behavior and suggesting ES as a powerful strategy to regulate osteogenesis.

ES-based therapies face multiple challenges, including a lack of standardized protocols, incomplete understanding of the specific molecular pathways and cellular responses to ES, and the necessity of ensuring safety, efficacy, and clinical viability. Future work must optimize ES parameters and unify methodologies, with interdisciplinary collaborations among bioengineers, molecular biologists, and clinicians, to address these barriers and achieve successful clinical translation.

## Figures and Tables

**Table 1:** Oral tissue-derived mesenchymal stem/stromal cell (OMSC) types and respective reported capacities to proliferate and differentiate in vitro into multiple lineages. Mesenchymal stem/stromal cell (MSC)-associated surface markers, and respective OMSC potential therapeutic applications are represented herein.

OMSC Type	In Vitro Multipotency	Proliferation Capacity Comparison	Expressed Cell Surface Markers	Dental/Periodontal Therapeutic Application	Reference
ABMSCs	Osteogenic, adipogenic, chondrogenic	Lower than DFPCs and PDLSCs	CD105, CD90, CD73, STRO-1	Defects in bone, regeneration of periodontal tissue	[33,38,39,40,41]
DFPCs	Osteogenic, adipogenic, chondrogenic, neurogenic, hepatogenic, cementogenic	Higher than DPSCs, ABMSCs, and SHED	CD13, CD29, CD44, CD56, CD73, CD90, CD105, CD146, CD271, STRO-1, NESTIN, NOTCH-1, HLA-ABC, HLA-I	Defects in bone, regeneration of tooth roots, regeneration of periodontal tissue	[32,42,43,44,45,46,47,48,49,50,51,52]
DPSCs	Osteo/odontogenic, adipogenic, chondrogenic, neurogenic, myogenic, cardiogenic, hepatogenic, and melanocyte differentiation	Lower than SHED, DFPCs, GMSCs, and SCAP	CD29, CD44, CD73, CD90, CD105, CD106, CD146, STRO-1	Defects in bone, repair of dentin–pulp	[30,34,41,43,44,48,53,54,55,56,57,58,59,60,61,62]
GMSCs	Osteogenic, adipogenic, chondrogenic, neurogenic, endoderm differentiation	Higher than DPSCs; lower than PDLSCs	CD13, CD29, CD44, CD73, CD90, CD105, CD146, STRO-1	Regeneration of periodontal tissue	[36,53,62,63,64,65,66,67,68,69]
PDLSCs	Osteo/odontogenic, adipogenic, chondrogenic, neurogenic, myogenic	Higher than ABMSCs and GMSCs	CD44, CD73, CD90, CD105, STRO-1	Regeneration of tooth roots, regeneration of periodontal tissue	[31,41,42,62,70,71,72,73,74,75]
SCAP	Osteo/odontogenic, adipogenic, neurogenic, hepatogenic	Higher than PDLSCs and DPSCs	CD13, CD24, CD29, CD44, CD49, CD51, CD56, CD61, CD73, CD90, CD105, CD106, CD146, CD166, STRO-1	Regeneration of bone, regeneration of tooth root, repair of dentin–pulp, regeneration of periodontal tissue	[34,48,57,76,77,78,79,80,81]
SHED	Osteo/odontogenic, adipogenic, chondrogenic, neurogenic, myogenic, angiogenic, hepatogenic	Higher than DPSCs; lower than DFPCs	CD29, CD44, CD73, CD90, CD105, CD146, CD166, STRO-1	Regeneration of tooth roots, repair of dentin–pulp	[43,45,47,60,63,76,82,83,84,85,86,87,88,89,90,91]
TGPCs	Osteo/odontogenic, adipogenic, chondrogenic, neurogenic, angiogenic, hepatogenic	DPSCs from tooth germs (TGPCs, at crown-completed stage) have a higher capacity than DPSCs from adults (at later stages of tooth development); higher than DFPCs	CD29, CD73, CD90, CD105, CD166	Regeneration of bone	[35,43,92,93,94,95,96,97,98]

ABMSC: alveolar bone-derived MSC; CD: cluster of differentiation; DFPC: dental follicle stem/progenitor cell; DPSC: dental pulp stem/stromal cell; GMSC: gingival MSC; HLA: Human Leukocyte Antigen; NESTIN: Neuroepithelial Stem Cell Protein; NOTCH-1: Neurogenic Locus Notch Homolog Protein 1; PDLSC: periodontal ligament stem/stromal cell; SCAP: stem/stromal cells from apical papilla; SHED: stem/stromal cells from human exfoliated deciduous teeth; STRO-1: Antigen-1; TGPC: tooth germ stem/progenitor cell.

## Data Availability

Not applicable.

## References

[1] Angelova Volponi A., Zaugg L.K., Neves V., Liu Y., Sharpe P.T. (2018). Tooth Repair and Regeneration. Curr. Oral Health Rep..

[2] Nanci A., Bosshardt D.D. (2006). Structure of Periodontal Tissues in Health and Disease. Periodontology 2000.

[3] Fehrenbach M.J., Popowics T. (2009). Illustrated Dental Embryology, Histology, and Anatomy E-Book.

[4] de Jong T., Bakker A.D., Everts V., Smit T.H. (2017). The Intricate Anatomy of the Periodontal Ligament and Its Development: Lessons for Periodontal Regeneration. J. Periodontal Res..

[5] Zhang X., Schuppan D., Becker J., Reichart P., Gelderblom H.R. (1993). Distribution of Undulin, Tenascin, and Fibronectin in the Human Periodontal Ligament and Cementum: Comparative Immunoelectron Microscopy with Ultra-Thin Cryosections. J. Histochem. Cytochem..

[6] Larjava H., Hakkinen L., Rahemtulla F. (1992). A Biochemical Analysis of Human Periodontal Tissue Proteoglycans. Biochem. J..

[7] Peres M.A., Macpherson L.M.D., Weyant R.J., Daly B., Venturelli R., Mathur M.R., Listl S., Celeste R.K., Guarnizo-Herreño C.C., Kearns C. (2019). Oral Diseases: A Global Public Health Challenge. Lancet.

[8] Righolt A.J., Jevdjevic M., Marcenes W., Listl S. (2018). Global-, Regional-, and Country-Level Economic Impacts of Dental Diseases in 2015. J. Dent. Res..

[9] Kassebaum N.J., Smith A.G.C., Bernabé E., Fleming T.D., Reynolds A.E., Vos T., Murray C.J.L., Marcenes W., Abyu G.Y., Alsharif U. (2017). Global, Regional, and National Prevalence, Incidence, and Disability-Adjusted Life Years for Oral Conditions for 195 Countries, 1990–2015: A Systematic Analysis for the Global Burden of Diseases, Injuries, and Risk Factors. J. Dent. Res..

[10] Jevdjevic M., Listl S. (2024). Global, Regional, and Country-Level Economic Impacts of Oral Conditions in 2019. J. Dent. Res..

[11] Nazir M.A. (2017). Prevalence of Periodontal Disease, Its Association with Systemic Diseases and Prevention. Int. J. Health Sci..

[12] Botelho J., Machado V., Leira Y., Proença L., Chambrone L., Mendes J.J. (2022). Economic Burden of Periodontitis in the United States and Europe: An Updated Estimation. J. Periodontol..

[13] Lin N.H., Gronthos S., Bartold P.M. (2008). Stem Cells and Periodontal Regeneration. Aust. Dent. J..

[14] Rams T.E., Degener J.E., van Winkelhoff A.J. (2014). Antibiotic Resistance in Human Chronic Periodontitis Microbiota. J. Periodontol..

[15] Haque M.M., Yerex K., Kelekis-Cholakis A., Duan K. (2022). Advances in Novel Therapeutic Approaches for Periodontal Diseases. BMC Oral Health.

[16] Herrera D., Sanz M., Jepsen S., Needleman I., Roldán S. (2002). A Systematic Review on the Effect of Systemic Antimicrobials as an Adjunct to Scaling and Root Planing in Periodontitis Patients. J. Clin. Periodontol..

[17] Narem R., Sambanis A. (1995). Tissue Engineering: From Biology to Biological Structures. Tissue Eng..

[18] Bartold P.M., McCulloch C.A.G., Narayanan A.S., Pitaru S. (2000). Tissue Engineering: A New Paradigm for Periodontal Regeneration Based on Molecular and Cell Biology. Periodontology.

[19] Lin W.-L., McCulloch C.A.G., Cho M.-I. (1994). Differentiation of Periodontal Ligament Fibroblasts into Osteoblasts during Socket Healing after Tooth Extraction in the Rat. Anat. Rec..

[20] Gould T.R.L. (1983). Ultrastructural Characteristics of Progenitor Cell Populations in the Periodontal Ligament. J. Dent. Res..

[21] Gould T.R.L., Melcher A.H., Brunette D.M. (1980). Migration and Division of Progenitor Cell Populations in Periodontal Ligament after Wounding. J. Periodontal Res..

[22] Levin M. (2014). Molecular Bioelectricity: How Endogenous Voltage Potentials Control Cell Behavior and Instruct Pattern Regulation In Vivo. Mol. Biol. Cell.

[23] Serena E., Figallo E., Tandon N., Cannizzaro C., Gerecht S., Elvassore N., Vunjak-Novakovic G. (2009). Electrical Stimulation of Human Embryonic Stem Cells: Cardiac Differentiation and the Generation of Reactive Oxygen Species. Exp. Cell Res..

[24] Eischen-Loges M., Oliveira K.M.C., Bhavsar M.B., Barker J.H., Leppik L. (2018). Pretreating Mesenchymal Stem Cells with Electrical Stimulation Causes Sustained Long-Lasting pro-Osteogenic Effects. PeerJ.

[25] Leppik L., Zhihua H., Mobini S., Thottakkattumana Parameswaran V., Eischen-Loges M., Slavici A., Helbing J., Pindur L., Oliveira K.M.C., Bhavsar M.B. (2018). Combining Electrical Stimulation and Tissue Engineering to Treat Large Bone Defects in a Rat Model. Sci. Rep..

[26] Mobini S., Leppik L., Parameswaran V.T., Barker J.H. (2017). In Vitro Effect of Direct Current Electrical Stimulation on Rat Mesenchymal Stem Cells. PeerJ.

[27] Wang X., Gao Y., Shi H., Liu N., Zhang W., Li H. (2016). Influence of the Intensity and Loading Time of Direct Current Electric Field on the Directional Migration of Rat Bone Marrow Mesenchymal Stem Cells. Front. Med..

[28] Yuan X., Arkonac D.E., Chao P.H.G., Vunjak-Novakovic G. (2014). Electrical Stimulation Enhances Cell Migration and Integrative Repair in the Meniscus. Sci. Rep..

[29] Zhang J., Li M., Kang E.T., Neoh K.G. (2016). Electrical Stimulation of Adipose-Derived Mesenchymal Stem Cells in Conductive Scaffolds and the Roles of Voltage-Gated Ion Channels. Acta Biomater..

[30] Gronthos S., Mankani M., Brahim J., Robey P.G., Shi S. (2000). Postnatal Human Dental Pulp Stem Cells (DPSC) In Vitro and In Vivo. Proc. Natl. Acad. Sci. USA.

[31] Seo B.M., Miura M., Gronthos S., Bartold P.M., Batouli S., Brahim J., Young M., Robey P.G., Wang C.Y., Shi S. (2004). Investigation of Multipotent Postnatal Stem Cells from Human Periodontal Ligament. Lancet.

[32] Morsczeck C., Götz W., Schierholz J., Zeilhofer F., Kühn U., Möhl C., Sippel C., Hoffmann K.H. (2005). Isolation of Precursor Cells (PCs) from Human Dental Follicle of Wisdom Teeth. Matrix Biol..

[33] Matsubara T., Suardita K., Ishii M., Sugiyama M., Igarashi A., Oda R., Nishimura M., Saito M., Nakagawa K., Yamanaka K. (2005). Alveolar Bone Marrow as a Cell Source for Regenerative Medicine: Differences Between Alveolar and Iliac Bone Marrow Stromal Cells. J. Bone Miner. Res..

[34] Sonoyama W., Liu Y., Fang D., Yamaza T., Seo B.M., Zhang C., Liu H., Gronthos S., Wang C.Y., Shi S. (2006). Mesenchymal Stem Cell-Mediated Functional Tooth Regeneration in Swine. PLoS ONE.

[35] Ikeda E., Yagi K., Kojima M., Yagyuu T., Ohshima A., Sobajima S., Tadokoro M., Katsube Y., Isoda K., Kondoh M. (2008). Multipotent Cells from the Human Third Molar: Feasibility of Cell-Based Therapy for Liver Disease. Differentiation.

[36] Zhang Q., Shi S., Liu Y., Uyanne J., Shi Y., Shi S., Le A.D. (2009). Mesenchymal Stem Cells Derived from Human Gingiva Are Capable of Immunomodulatory Functions and Ameliorate Inflammation-Related Tissue Destruction in Experimental Colitis. J. Immunol..

[37] Liu J., Yu F., Sun Y., Jiang B., Zhang W., Yang J., Xu G.T., Liang A., Liu S. (2015). Concise Reviews: Characteristics and Potential Applications of Human Dental Tissue-Derived Mesenchymal Stem Cells. Stem Cells.

[38] Park J.C., Kim J.C., Kim Y.T., Choi S.H., Cho K.S., Im G., Kim B.S., Kim C.S. (2012). Acquisition of Human Alveolar Bone-Derived Stromal Cells Using Minimally Irrigated Implant Osteotomy: In Vitro and In Vivo Evaluations. J. Clin. Periodontol..

[39] Pekovits K., Kröpfl J.M., Stelzer I., Payer M., Hutter H., Dohr G. (2013). Human Mesenchymal Progenitor Cells Derived from Alveolar Bone and Human Bone Marrow Stromal Cells: A Comparative Study. Histochem. Cell Biol..

[40] Mason S., Tarle S.A., Osibin W., Kinfu Y., Kaigler D. (2013). Standardization and Safety of Alveolar Bone–Derived Stem Cell Isolation. J. Dent. Res..

[41] Wang L., Shen H., Zheng W., Tang L., Yang Z., Gao Y., Yang Q., Wang C., Duan Y., Jin Y. (2010). Characterization of Stem Cells from Alveolar Periodontal Ligament. Tissue Eng. Part A.

[42] Luan X., Ito Y., Dangaria S., Diekwisch T.G.H. (2006). Dental Follicle Progenitor Cell Heterogeneity in the Developing Mouse Periodontium. Stem Cells Dev..

[43] Zhou T., Pan J., Wu P., Huang R., Du W., Zhou Y., Wan M., Fan Y., Xu X., Zhou X. (2019). Dental Follicle Cells: Roles in Development and Beyond. Stem Cells Int..

[44] Shoi K., Aoki K., Ohya K., Takagi Y., Shimokawa H. (2014). Characterization of Pulp and Follicle Stem Cells from Impacted Supernumerary Maxillary Incisors. Pediatr. Dent..

[45] Yildirim S., Zibandeh N., Genc D., Ozcan E.M., Goker K., Akkoc T. (2016). The Comparison of the Immunologic Properties of Stem Cells Isolated from Human Exfoliated Deciduous Teeth, Dental Pulp, and Dental Follicles. Stem Cells Int..

[46] Kémoun P., Laurencin-Dalicieux S., Rue J., Farges J.C., Gennero I., Conte-Auriol F., Briand-Mesange F., Gadelorge M., Arzate H., Narayanan A.S. (2007). Human Dental Follicle Cells Acquire Cementoblast Features under Stimulation by BMP-2/-7 and Enamel Matrix Derivatives (EMD) In Vitro. Cell Tissue Res..

[47] Völlner F., Ernst W., Driemel O., Morsczeck C. (2009). A Two-Step Strategy for Neuronal Differentiation In Vitro of Human Dental Follicle Cells. Differentiation.

[48] Patil R., Kumar B.M., Lee W.J., Jeon R.H., Jang S.J., Lee Y.M., Park B.W., Byun J.H., Ahn C.S., Kim J.W. (2014). Multilineage Potential and Proteomic Profiling of Human Dental Stem Cells Derived from a Single Donor. Exp. Cell Res..

[49] Jo Y.Y., Lee H.J., Kook S.Y., Choung H.W., Park J.Y., Chung J.H., Choung Y.H., Kim E.S., Yang H.C., Choung P.H. (2007). Isolation and Characterization of Postnatal Stem Cells from Human Dental Tissues. Tissue Eng..

[50] Honda M.J., Imaizumi M., Tsuchiya S., Morsczeck C. (2010). Dental Follicle Stem Cells and Tissue Engineering. J. Oral Sci..

[51] Mori G., Ballini A., Carbone C., Oranger A., Brunetti G., di Benedetto A., Rapone B., Cantore S., Di Comite M., Colucci S. (2012). Osteogenic Differentiation of Dental Follicle Stem Cells. Int. J. Med. Sci..

[52] Bi R., Lyu P., Song Y., Li P., Song D., Cui C., Fan Y. (2021). Function of Dental Follicle Progenitor/Stem Cells and Their Potential in Regenerative Medicine: From Mechanisms to Applications. Biomolecules.

[53] Angelopoulos I., Brizuela C., Khoury M. (2018). Gingival Mesenchymal Stem Cells Outperform Haploidentical Dental Pulp-Derived Mesenchymal Stem Cells in Proliferation Rate, Migration Ability, and Angiogenic Potential. Cell Transplant..

[54] Gronthos S., Brahim J., Li W., Fisher L.W., Cherman N., Boyde A., DenBesten P., Robey P.G., Shi S. (2002). Stem Cell Properties of Human Dental Pulp Stem Cells. J. Dent. Res..

[55] Laino G., d’Aquino R., Graziano A., Lanza V., Carinci F., Naro F., Pirozzi G., Papaccio G. (2005). A New Population of Human Adult Dental Pulp Stem Cells: A Useful Source of Living Autologous Fibrous Bone Tissue (LAB). J. Bone Miner. Res..

[56] d’Aquino R., Graziano A., Sampaolesi M., Laino G., Pirozzi G., De Rosa A., Papaccio G. (2007). Human Postnatal Dental Pulp Cells Co-Differentiate into Osteoblasts and Endotheliocytes: A Pivotal Synergy Leading to Adult Bone Tissue Formation. Cell Death Differ..

[57] Armiñán A., Gandía C., Bartual M., García-Verdugo J.M., Lledó E., Mirabet V., Llop M., Barea J., Montero J.A., Sepúlveda P. (2009). Cardiac Differentiation Is Driven by NKX2. 5 and GATA4 Nuclear Translocation in Tissue-Specific Mesenchymal Stem Cells. Stem Cells Dev..

[58] Arthur A., Rychkov G., Shi S., Koblar S.A., Gronthos S. (2008). Adult Human Dental Pulp Stem Cells Differentiate Toward Functionally Active Neurons Under Appropriate Environmental Cues. Stem Cells.

[59] Stevens A., Zuliani T., Olejnik C., LeRoy H., Obriot H., Kerr-Conte J., Formstecher P., Bailliez Y., Polakowska R.R. (2008). Human Dental Pulp Stem Cells Differentiate into Neural Crest-Derived Melanocytes and Have Label-Retaining and Sphere-Forming Abilities. Stem Cells Dev..

[60] Nakamura S., Yamada Y., Katagiri W., Sugito T., Ito K., Ueda M. (2009). Stem Cell Proliferation Pathways Comparison between Human Exfoliated Deciduous Teeth and Dental Pulp Stem Cells by Gene Expression Profile from Promising Dental Pulp. J. Endod..

[61] Sloan A.J., Waddington R.J. (2009). Dental Pulp Stem Cells: What, Where, How?. Int. J. Paediatr. Dent..

[62] Assem M., Kamal S., Sabry D., Soliman N., Aly R.M. (2018). Preclinical Assessment of the Proliferation Capacity of Gingival and Periodontal Ligament Stem Cells from Diabetic Patients. Open Access Maced. J. Med. Sci..

[63] Tang L., Li N., Xie H., Jin Y. (2011). Characterization of Mesenchymal Stem Cells from Human Normal and Hyperplastic Gingiva. J. Cell Physiol..

[64] Wang F., Yu M., Yan X., Wen Y., Zeng Q., Yue W., Yang P., Pei X. (2011). Gingiva-Derived Mesenchymal Stem Cell-Mediated Therapeutic Approach for Bone Tissue Regeneration. Stem Cells Dev..

[65] Marynka-Kalmani K., Treves S., Yafee M., Rachima H., Gafni Y., Cohen M.A., Pitaru S. (2010). The Lamina Propria of Adult Human Oral Mucosa Harbors a Novel Stem Cell Population. Stem Cells.

[66] Mitrano T.I., Grob M.S., Carrión F., Nova-Lamperti E., Luz P.A., Fierro F.S., Quintero A., Chaparro A., Sanz A. (2010). Culture and Characterization of Mesenchymal Stem Cells From Human Gingival Tissue. J. Periodontol..

[67] Van Pham P., Tran N.Y., Phan N.L.C., Vu N.B., Phan N.K. (2016). Vitamin C Stimulates Human Gingival Stem Cell Proliferation and Expression of Pluripotent Markers. In Vitro Cell Dev. Biol. Anim..

[68] Fawzy El-Sayed K.M., Dörfer C.E. (2016). Gingival Mesenchymal Stem/Progenitor Cells: A Unique Tissue Engineering Gem. Stem Cells Int..

[69] Chen X., Chen Y., Hou Y., Song P., Zhou M., Nie M., Liu X. (2019). Modulation of Proliferation and Differentiation of Gingiva-derived Mesenchymal Stem Cells by Concentrated Growth Factors: Potential Implications in Tissue Engineering for Dental Regeneration and Repair. Int. J. Mol. Med..

[70] Shi S., Miura M., Seo B.M., Robey P.G., Bartold P.M., Gronthos S. (2005). The Efficacy of Mesenchymal Stem Cells to Regenerate and Repair Dental Structures. Orthod. Craniofac. Res..

[71] Xu J., Wang W., Kapila Y., Lotz J., Kapila S. (2008). Multiple Differentiation Capacity of STRO-1+/CD146+ PDL Mesenchymal Progenitor Cells. Stem Cells Dev..

[72] Zhu W., Liang M. (2015). Periodontal Ligament Stem Cells: Current Status, Concerns, and Future Prospects. Stem Cells Int..

[73] Nagatomo K., Komaki M., Sekiya I., Sakaguchi Y., Noguchi K., Oda S., Muneta T., Ishikawa I. (2006). Stem Cell Properties of Human Periodontal Ligament Cells. J. Periodontal Res..

[74] Zheng W., Wang S., Wang J., Jin F. (2015). Periodontitis Promotes the Proliferation and Suppresses the Differentiation Potential of Human Periodontal Ligament Stem Cells. Int. J. Mol. Med..

[75] Liu Y., Zheng Y., Ding G., Fang D., Zhang C., Bartold P.M., Gronthos S., Shi S., Wang S. (2008). Periodontal Ligament Stem Cell-Mediated Treatment for Periodontitis in Miniature Swine. Stem Cells.

[76] Miura M., Gronthos S., Zhao M., Lu B., Fisher L.W., Robey P.G., Shi S. (2003). SHED: Stem Cells from Human Exfoliated Deciduous Teeth. Proc. Natl. Acad. Sci. USA.

[77] Chen K., Xiong H., Huang Y., Liu C. (2013). Comparative Analysis of in Vitro Periodontal Characteristics of Stem Cells from Apical Papilla (SCAP) and Periodontal Ligament Stem Cells (PDLSCs). Arch. Oral Biol..

[78] Abe S., Yamaguchi S., Amagasa T. (2007). Multilineage Cells from Apical Pulp of Human Tooth with Immature Apex. Oral Sci. Int..

[79] Sonoyama W., Liu Y., Yamaza T., Tuan R.S., Wang S., Shi S., Huang G.T.J. (2008). Characterization of the Apical Papilla and Its Residing Stem Cells from Human Immature Permanent Teeth: A Pilot Study. J. Endod..

[80] Wang J., Liu B., Gu S., Liang J. (2012). Effects of Wnt/β-Catenin Signalling on Proliferation and Differentiation of Apical Papilla Stem Cells. Cell Prolif..

[81] Kang J., Fan W., Deng Q., He H., Huang F. (2019). Stem Cells from the Apical Papilla: A Promising Source for Stem Cell-Based Therapy. BioMed Res. Int..

[82] Wang X., Sha X.J., Li G.H., Yang F.S., Ji K., Wen L.Y., Liu S.Y., Chen L., Ding Y., Xuan K. (2012). Comparative Characterization of Stem Cells from Human Exfoliated Deciduous Teeth and Dental Pulp Stem Cells. Arch. Oral Biol..

[83] Suchánek J., Visek B., Soukup T., El-Din Mohamed S.K., Ivancakova R., Mokry J., Aboul-Ezz E.H.A., Omran A. (2010). Stem Cells from Human Exfoliated Deciduous Teeth—Isolation, Long Term Cultivation and Phenotypical Analysis. Acta Med..

[84] Kunimatsu R., Nakajima K., Awada T., Tsuka Y., Abe T., Ando K., Hiraki T., Kimura A., Tanimoto K. (2018). Comparative Characterization of Stem Cells from Human Exfoliated Deciduous Teeth, Dental Pulp, and Bone Marrow–Derived Mesenchymal Stem Cells. Biochem. Biophys. Res. Commun..

[85] Kerkis I., Kerkis A., Dozortsev D., Stukart-Parsons G.C., Gomes Massironi S.M., Pereira L.V., Caplan A.I., Cerruti H.F. (2007). Isolation and Characterization of a Population of Immature Dental Pulp Stem Cells Expressing OCT-4 and Other Embryonic Stem Cell Markers. Cells Tissues Organs.

[86] Cordeiro M.M., Dong Z., Kaneko T., Zhang Z., Miyazawa M., Shi S., Smith A.J., Nör J.E. (2008). Dental Pulp Tissue Engineering with Stem Cells from Exfoliated Deciduous Teeth. J. Endod..

[87] Ishkitiev N., Yaegaki K., Calenic B., Nakahara T., Ishikawa H., Mitiev V., Haapasalo M. (2010). Deciduous and Permanent Dental Pulp Mesenchymal Cells Acquire Hepatic Morphologic and Functional Features In Vitro. J. Endod..

[88] Tsutsui T.W. (2020). Dental Pulp Stem Cells: Advances to Applications. Stem Cells Cloning.

[89] Zhang X., Lei T., Chen P., Wang L., Wang J., Wang D., Guo W., Zhou Y., Li Q., Du H. (2021). Stem Cells from Human Exfoliated Deciduous Teeth Promote Hair Regeneration in Mouse. Cell Transplant..

[90] Annibali S., Cristalli M.P., Tonoli F., Polimeni A. (2014). Stem Cells Derived from Human Exfoliated Deciduous Teeth: A Narrative Synthesis of Literature. Eur. Rev. Med. Pharmacol. Sci..

[91] Yang X., Ma Y., Guo W., Yang B., Tian W. (2019). Stem Cells from Human Exfoliated Deciduous Teeth as an Alternative Cell Source in Bio-Root Regeneration. Theranostics.

[92] Takeda T., Tezuka Y., Horiuchi M., Hosono K., Iida K., Hatakeyama D., Miyaki S., Kunisada T., Shibata T., Tezuka K. (2008). Characterization of Dental Pulp Stem Cells of Human Tooth Germs. J. Dent. Res..

[93] Yalvac M.E., Ramazanoglu M., Rizvanov A.A., Sahin F., Bayrak O.F., Salli U., Palotás A., Kose G.T. (2010). Isolation and Characterization of Stem Cells Derived from Human Third Molar Tooth Germs of Young Adults: Implications in Neo-Vascularization, Osteo-, Adipo- and Neurogenesis. Pharmacogenomics J..

[94] Yalvac M.E., Ramazanoglu M., Gumru O.Z., Sahin F., Palotás A., Rizvanov A.A. (2009). Comparison and Optimisation of Transfection of Human Dental Follicle Cells, a Novel Source of Stem Cells, with Different Chemical Methods and Electro-Poration. Neurochem. Res..

[95] Yalvac M.E., Ramazanoglu M., Tekguc M., Bayrak O.F., Shafigullina A.K., Salafutdinov I.I., Blatt N.L., Kiyasov A.P., Sahin F., Palotas A. (2010). Human Tooth Germ Stem Cells Preserve Neuro-Protective Effects after Long-Term Cryo-Preservation. Curr. Neurovasc. Res..

[96] Yalvaç M.E., Yilmaz A., Mercan D., Aydin S., Dogan A., Arslan A., Demir Z., Salafutdinov I.I., Shafigullina A.K., Sahin F. (2011). Differentiation and Neuro-Protective Properties of Immortalized Human Tooth Germ Stem Cells. Neurochem. Res..

[97] Doǧan A., Yalvaç M.E., Şahin F., Kabanov A.V., Palotás A., Rizvanov A.A. (2012). Differentiation of Human Stem Cells Is Promoted by Amphiphilic Pluronic Block Copolymers. Int. J. Nanomed..

[98] Guven E.P., Yalvac M.E., Sahin F., Yazici M.M., Rizvanov A.A., Bayirli G. (2011). Effect of Dental Materials Calcium Hydroxide–Containing Cement, Mineral Trioxide Aggregate, and Enamel Matrix Derivative on Proliferation and Differentiation of Human Tooth Germ Stem Cells. J. Endod..

[99] Yamada Y., Ito K., Nakamura S., Ueda M., Nagasaka T. (2011). Promising Cell-Based Therapy for Bone Regeneration Using Stem Cells from Deciduous Teeth, Dental Pulp, and Bone Marrow. Cell Transplant..

[100] Yamada Y., Nakamura S., Ito K., Sugito T., Yoshimi R., Nagasaka T., Ueda M. (2010). A Feasibility of Useful Cell-Based Therapy by Bone Regeneration with Deciduous Tooth Stem Cells, Dental Pulp Stem Cells, or Bone-Marrow-Derived Mesenchymal Stem Cells for Clinical Study Using Tissue Engineering Technology. Tissue Eng. Part A.

[101] Hu J., Cao Y., Xie Y., Wang H., Fan Z., Wang J., Zhang C., Wang J., Wu C.T., Wang S. (2016). Periodontal Regeneration in Swine after Cell Injection and Cell Sheet Transplantation of Human Dental Pulp Stem Cells Following Good Manufacturing Practice. Stem Cell Res. Ther..

[102] Santos M.S., Carvalho M.S., Silva J.C. (2023). Recent Advances on Electrospun Nanofibers for Periodontal Regeneration. Nanomaterials.

[103] Ding G., Liu Y., Wang W., Wei F., Liu D., Fan Z., An Y., Zhang C., Wang S. (2010). Allogeneic Periodontal Ligament Stem Cell Therapy for Periodontitis in Swine. Stem Cells.

[104] Chen F.M., Gao L.N., Tian B.M., Zhang X.Y., Zhang Y.J., Dong G.Y., Lu H., Chu Q., Xu J., Yu Y. (2016). Treatment of Periodontal Intrabony Defects Using Autologous Periodontal Ligament Stem Cells: A Randomized Clinical Trial. Stem Cell Res. Ther..

[105] Heng W., Bhavsar M., Han Z., Barker J.H. (2020). Effects of Electrical Stimulation on Stem Cells. Curr. Stem Cell Res. Ther..

[106] Sun S., Titushkin I., Cho M. (2006). Regulation of Mesenchymal Stem Cell Adhesion and Orientation in 3D Collagen Scaffold by Electrical Stimulus. Bioelectrochemistry.

[107] Radisic M., Park H., Shing H., Consi T., Schoen F.J., Langer R., Freed L.E., Vunjak-Novakovic G. (2004). Functional Assembly of Engineered Myocardium by Electrical Stimulation of Cardiac Myocytes Cultured on Scaffolds. Proc. Natl. Acad. Sci. USA.

[108] Balint R., Cassidy N.J., Cartmell S.H. (2012). Electrical Stimulation: A Novel Tool for Tissue Engineering. Tissue Eng. Part B Rev..

[109] Fukada E., Yasuda I. (1957). On the Piezoelectric Effect of Bone. J. Physical Soc. Jpn..

[110] Ahn A.C., Grodzinsky A.J. (2009). Relevance of Collagen Piezoelectricity to “Wolff’s Law”: A Critical Review. Med. Eng. Phys..

[111] Chen C., Bai X., Ding Y., Lee I.S. (2019). Electrical Stimulation as a Novel Tool for Regulating Cell Behavior in Tissue Engineering. Biomater. Res..

[112] Guillot-Ferriols M., Lanceros-Méndez S., Gómez Ribelles J.L., Gallego Ferrer G. (2022). Electrical Stimulation: Effective Cue to Direct Osteogenic Differentiation of Mesenchymal Stem Cells?. Biomater. Adv..

[113] Hronik-Tupaj M., Rice W.L., Cronin-Golomb M., Kaplan D.L., Georgakoudi I. (2011). Osteoblastic Differentiation and Stress Response of Human Mesenchymal Stem Cells Exposed to Alternating Current Electric Fields. Biomed. Eng. Online.

[114] Silva J.C., Meneses J., Garrudo F.F.F., Fernandes S.R., Alves N., Ferreira F.C., Pascoal-Faria P. (2024). Direct Coupled Electrical Stimulation towards Improved Osteogenic Differentiation of Human Mesenchymal Stem/Stromal Cells: A Comparative Study of Different Protocols. Sci. Rep..

[115] Hu W.W., Chen T.C., Tsao C.W., Cheng Y.C. (2019). The Effects of Substrate-Mediated Electrical Stimulation on the Promotion of Osteogenic Differentiation and Its Optimization. J. Biomed. Mater. Res. B Appl. Biomater..

[116] Chang K.A., Kim J.W., Kim J.A., Lee S., Kim S., Suh W.H., Kim H.S., Kwon S., Kim S.J., Suh Y.H. (2011). Biphasic Electrical Currents Stimulation Promotes Both Proliferation and Differentiation of Fetal Neural Stem Cells. PLoS ONE.

[117] Young Park S., Park J., Hyun Sim S., Gyu Sung M., Kim K.S., Hee Hong B., Hong S., Hong S., Park S.Y., Sim S.H. (2011). Enhanced Differentiation of Human Neural Stem Cells into Neurons on Graphene. Adv. Mater..

[118] Du J., Zhen G., Chen H., Zhang S., Qing L., Yang X., Lee G., Mao H.Q., Jia X. (2018). Optimal Electrical Stimulation Boosts Stem Cell Therapy in Nerve Regeneration. Biomaterials.

[119] Genovese J.A., Spadaccio C., Chachques E., Schussler O., Carpentier A., Chachques J.C., Patel A.N. (2009). Cardiac Pre-Differentiation of Human Mesenchymal Stem Cells by Electrostimulation. Front. Biosci..

[120] Mooney E., Mackle J.N., Blond D.J.P., O’Cearbhaill E., Shaw G., Blau W.J., Barry F.P., Barron V., Murphy J.M. (2012). The Electrical Stimulation of Carbon Nanotubes to Provide a Cardiomimetic Cue to MSCs. Biomaterials.

[121] Pietronave S., Zamperone A., Oltolina F., Colangelo D., Follenzi A., Novelli E., Diena M., Pavesi A., Consolo F., Fiore G.B. (2013). Monophasic and Biphasic Electrical Stimulation Induces a Precardiac Differentiation in Progenitor Cells Isolated from Human Heart. Stem Cells Dev..

[122] Pavesi A., Soncini M., Zamperone A., Pietronave S., Medico E., Redaelli A., Prat M., Fiore G.B. (2014). Electrical Conditioning of Adipose-Derived Stem Cells in a Multi-Chamber Culture Platform. Biotechnol. Bioeng..

[123] Sauer H., Rahimi G., Hescheler J., Wartenberg M. (1999). Effects of Electrical Fields on Cardiomyocyte Differentiation of Embryonic Stem Cells. J. Cell. Biochem..

[124] Hernández D., Millard R., Sivakumaran P., Wong R.C.B., Crombie D.E., Hewitt A.W., Liang H., Hung S.S.C., Pébay A., Shepherd R.K. (2016). Electrical Stimulation Promotes Cardiac Differentiation of Human Induced Pluripotent Stem Cells. Stem Cells Int..

[125] Mardani M., Roshankhah S., Hashemibeni B., Salahshoor M., Naghsh E., Esfandiari E. (2016). Induction of Chondrogenic Differentiation of Human Adipose-Derived Stem Cells by Low Frequency Electric Field. Adv. Biomed. Res..

[126] Kwon H.J., Lee G.S., Chun H. (2016). Electrical Stimulation Drives Chondrogenesis of Mesenchymal Stem Cells in the Absence of Exogenous Growth Factors. Sci. Rep..

[127] Esfandiari E., Roshankhah S., Mardani M., Hashemibeni B., Naghsh E., Kazemi M., Salahshoor M. (2014). The Effect of High Frequency Electric Field on Enhancement of Chondrogenesis in Human Adipose-Derived Stem Cells. Iran J. Basic Med. Sci..

[128] Vaca-González J.J., Clara-Trujillo S., Guillot-Ferriols M., Ródenas-Rochina J., Sanchis M.J., Ribelles J.L.G., Garzón-Alvarado D.A., Ferrer G.G. (2020). Effect of Electrical Stimulation on Chondrogenic Differentiation of Mesenchymal Stem Cells Cultured in Hyaluronic Acid—Gelatin Injectable Hydrogels. Bioelectrochemistry.

[129] Bai H., Forrester J.V., Zhao M. (2011). DC Electric Stimulation Upregulates Angiogenic Factors in Endothelial Cells Through Activation of VEGF Receptors. Cytokine.

[130] Ud-Din S., Sebastian A., Giddings P., Colthurst J., Whiteside S., Morris J., Nuccitelli R., Pullar C., Baguneid M., Bayat A. (2015). Angiogenesis Is Induced and Wound Size Is Reduced by Electrical Stimulation in an Acute Wound Healing Model in Human Skin. PLoS ONE.

[131] Beugels J., Molin D.G.M., Ophelders D.R.M.G., Rutten T., Kessels L., Kloosterboer N., de Grzymala A.A.P., Kramer B.W.W., van der Hulst R.R.W.J., Wolfs T.G.A.M. (2019). Electrical Stimulation Promotes the Angiogenic Potential of Adipose-Derived Stem Cells. Sci. Rep..

[132] Saghiri M.A., Shekarian M., Samadi F., Briss D.S., Napoli S., Conte M. (2025). The Impact of PH on the Piezoelectric Properties of Dentin in Root Canal Treated Teeth: Implications for Dental Materials and Oral Health. J. Endod..

[133] Ghosh S., Qiao W., Yang Z., Orrego S., Neelakantan P. (2023). Engineering Dental Tissues Using Biomaterials with Piezoelectric Effect: Current Progress and Future Perspectives. J. Funct. Biomater..

[134] Braden M., Bairstow A.G., Beider I., Ritter B.G. (1966). Electrical and Piezo-Electrical Properties of Dental Hard Tissues. Nature.

[135] Marino A.A., Gross B.D. (1989). Piezoelectricity in Cementum, Dentine and Bone. Arch. Oral Biol..

[136] Reyes-Gasga J., Galindo-Mentle M., Brès E., Vargas-Becerril N., Orozco E., Rodríguez-Gómez A., García-García R. (2020). Detection of the Piezoelectricity Effect in Nanocrystals from Human Teeth. J. Phys. Chem. Solids.

[137] Athenstaedt H. (1971). Pyroelectric and Piezoelectric Behaviour of Human Dental Hard Tissues. Arch. Oral Biol..

[138] Wang T., Feng Z., Song Y., Chen X. (2007). Piezoelectric Properties of Human Dentin and Some Influencing Factors. Dent. Mater..

[139] Križaj D., Jan J., Valenčič V. (2004). Modeling AC Current Conduction Through a Human Tooth. Bioelectromagnetics.

[140] Reyes-Gasga J., García G.R., Alvarez-Fregoso O., Chávez-Carvayar J.A., Vargas-Ulloa L.E. (1999). Conductivity in Human Tooth Enamel. J. Mater. Sci..

[141] Nekoofar M.H., Ghandi M.M., Hayes S.J., Dummer P.M.H. (2006). The Fundamental Operating Principles of Electronic Root Canal Length Measurement Devices. Int. Endod. J..

[142] Drolshagen M., Keilig L., Hasan I., Reimann S., Deschner J., Brinkmann K.T., Krause R., Favino M., Bourauel C. (2011). Development of a Novel Intraoral Measurement Device to Determine the Biomechanical Characteristics of the Human Periodontal Ligament. J. Biomech..

[143] Jin Y., Li J., Wang Y., Ye R., Feng X., Jing Z., Zhao Z. (2015). Functional Role of Mechanosensitive Ion Channel Piezo1 in Human Periodontal Ligament Cells. Angle Orthod..

[144] Kaynak D., Meffert R., Günhan M., Mer Günhan Ö. (2005). A Histopathologic Investigation on the Effects of Electrical Stimulation on Periodontal Tissue Regeneration in Experimental Bony Defects in Dogs. J. Periodontol..

[145] Cosoli G., Scalise L., Cerri G., Russo P., Tricarico G., Tomasini E.P. (2017). Bioimpedancemetry for the Assessment of Periodontal Tissue Inflammation: A Numerical Feasibility Study. Comput. Methods Biomech. Biomed. Eng..

[146] Thrivikraman G., Boda S.K., Basu B. (2018). Unraveling the Mechanistic Effects of Electric Field Stimulation towards Directing Stem Cell Fate and Function: A Tissue Engineering Perspective. Biomaterials.

[147] Xiong G.M., Do A.T., Wang J.K., Yeoh C.L., Yeo K.S., Choong C. (2015). Development of a Miniaturized Stimulation Device for Electrical Stimulation of Cells. J. Biol. Eng..

[148] Srirussamee K., Mobini S., Cassidy N.J., Cartmell S.H. (2019). Direct Electrical Stimulation Enhances Osteogenesis by Inducing Bmp2 and Spp1 Expressions from Macrophages and Preosteoblasts. Biotechnol. Bioeng..

[149] Mobini S., Leppik L., Barker J.H. (2016). Direct Current Electrical Stimulation Chamber for Treating Cells in Vitro. Biotechniques.

[150] Nguyen H.T., Wei C., Chow J.K., Nguy L., Nguyen H.K., Schmidt C.E. (2013). Electric Field Stimulation through a Substrate Influences Schwann Cell and Extracellular Matrix Structure. J. Neural Eng..

[151] Bizios R., Ullmann K.R., Supronowicz P.R., Ajayan P.M., Metzger D.W., Arulanandam B.P. (2002). Novel Current-Conducting Composite Substrates for Exposing Osteoblasts to Alternating Current Stimulation. J. Biomed. Mater. Res..

[152] Hartig M., Joos U., Wiesmann H.P. (2000). Capacitively Coupled Electric Fields Accelerate Proliferation of Osteoblast-like Primary Cells and Increase Bone Extracellular Matrix Formation in Vitro. Eur. Biophys. J..

[153] Griffin M., Iqbal S.A., Sebastian A., Colthurst J., Bayat A. (2011). Degenerate Wave and Capacitive Coupling Increase Human MSC Invasion and Proliferation While Reducing Cytotoxicity in an In Vitro Wound Healing Model. PLoS ONE.

[154] Pickering S.A.W., Scammell B.E. (2002). Electromagnetic Fields for Bone Healing. Int. J. Low Extrem. Wounds.

[155] Kotnik T., Miklavčič D. (2006). Theoretical Evaluation of Voltage Inducement on Internal Membranes of Biological Cells Exposed to Electric Fields. Biophys. J..

[156] Ross C.L., Siriwardane M., Almeida-Porada G., Porada C.D., Brink P., Christ G.J., Harrison B.S. (2015). The Effect of Low-Frequency Electromagnetic Field on Human Bone Marrow Stem/Progenitor Cell Differentiation. Stem Cell Res..

[157] McCullen S.D., McQuilling J.P., Grossfeld R.M., Lubischer J.L., Clarke L.I., Loboa E.G. (2010). Application of Low-Frequency Alternating Current Electric Fields Via Interdigitated Electrodes: Effects on Cellular Viability, Cytoplasmic Calcium, and Osteogenic Differentiation of Human Adipose-Derived Stem Cells. Tissue Eng. Part C Methods.

[158] Yuan Y., Zheng L., Feng Z., Yang G. (2021). Different Effects of Monophasic Pulses and Biphasic Pulses Applied by a Bipolar Stimulation Electrode in the Rat Hippocampal CA1 Region. Biomed. Eng. Online.

[159] Babona-Pilipos R., Pritchard-Oh A., Popovic M.R., Morshead C.M. (2015). Biphasic Monopolar Electrical Stimulation Induces Rapid and Directed Galvanotaxis in Adult Subependymal Neural Precursors. Stem Cell Res. Ther..

[160] Field-Fote E.C., Anderson B., Robertson V.J., Spielholz N.I. (2003). Monophasic and Biphasic Stimulation Evoke Different Responses. Muscle Nerve.

[161] Emmanuel B.S. A Study of the Effectiveness of Monophasic Electrical Stimulation in Enhancing Neuromuscular Tissue Function. Proceedings of the 5th International Conference on Information Technology for Education and Development (ITED 2022).

[162] Grill W.M. (2015). Model-Based Analysis and Design of Waveforms for Efficient Neural Stimulation. Prog. Brain Res..

[163] Wongsarnpigoon A., Woock J.P., Grill W.M. (2010). Efficiency Analysis of Waveform Shape for Electrical Excitation of Nerve Fibers. IEEE Trans. Neural Syst. Rehabil. Eng..

[164] Foutz T.J., McIntyre C.C. (2010). Evaluation of Novel Stimulus Waveforms for Deep Brain Stimulation. J. Neural Eng..

[165] Hsu G., Farahani F., Parra L.C. (2021). Cutaneous Sensation of Electrical Stimulation Waveforms. Brain Stimul..

[166] Uemura M., Sugimoto M., Yoshijawa Y., Hiramatsu T., Inoue T. (2021). Monophasic Pulsed Current Stimulation of Duty Cycle 10% Promotes Differentiation of Human Dermal Fibroblasts into Myofibroblasts. Phys. Ther. Res..

[167] Dubey A.K., Gupta S.D., Basu B. (2011). Optimization of Electrical Stimulation Parameters for Enhanced Cell Proliferation on Biomaterial Surfaces. J. Biomed. Mater. Res. B Appl. Biomater..

[168] Sommer M., Kamm T., Tergau F., Ulm G., Paulus W. (2002). Repetitive Paired-Pulse Transcranial Magnetic Stimulation Affects Corticospinal Excitability and Finger Tapping in Parkinson’s Disease. Clin. Neurophysiol..

[169] Waataja J.J., Tweden K.S., Honda C.N. (2011). Effects of High-Frequency Alternating Current on Axonal Conduction through the Vagus Nerve. J. Neural Eng..

[170] Kim J., Yang H.J., Cho T.H., Lee S.E., Park Y.D., Kim H.M., Kim I.S., Seo Y.K., Hwang S.J., Kim S.J. (2013). Enhanced Regeneration of Rabbit Mandibular Defects through a Combined Treatment of Electrical Stimulation and RhBMP-2 Application. Med. Biol. Eng. Comput..

[171] Hess R., Jaeschke A., Neubert H., Hintze V., Moeller S., Schnabelrauch M., Wiesmann H.P., Hart D.A., Scharnweber D. (2012). Synergistic Effect of Defined Artificial Extracellular Matrices and Pulsed Electric Fields on Osteogenic Differentiation of Human MSCs. Biomaterials.

[172] Nuccitelli R., Lui K., Kreis M., Athos B., Nuccitelli P. (2013). Nanosecond Pulsed Electric Field Stimulation of Reactive Oxygen Species in Human Pancreatic Cancer Cells Is Ca^2+^-Dependent. Biochem. Biophys. Res. Commun..

[173] Tzoneva R. (2014). Influence of Electric Field on Cell Behavior. Electrotreatment of Cells for Biomedical Applications. Asian J. Phys..

[174] Khaw J.S., Xue R., Cassidy N.J., Cartmell S.H. (2022). Electrical Stimulation of Titanium to Promote Stem Cell Orientation, Elongation and Osteogenesis. Acta Biomater..

[175] Ravikumar K., Boda S.K., Basu B. (2017). Synergy of Substrate Conductivity and Intermittent Electrical Stimulation towards Osteogenic Differentiation of Human Mesenchymal Stem Cells. Bioelectrochemistry.

[176] Yadollahpour A., Jalilifar M. (2014). Electromagnetic Fields in the Treatment of Wound: A Review of Current Techniques and Future Perspective. J. Pure Appl. Microbiol..

[177] Zhu R., Sun Z., Li C., Ramakrishna S., Chiu K., He L. (2019). Electrical Stimulation Affects Neural Stem Cell Fate and Function In Vitro. Exp. Neurol..

[178] Aguilar A.A., Ho M.C., Chang E., Carlson K.W., Natarajan A., Marciano T., Bomzon Z., Patel C.B. (2021). Permeabilizing Cell Membranes with Electric Fields. Cancers.

[179] Haddad J.B., Obolensky A.G., Shinnick P. (2007). The Biologic Effects and the Therapeutic Mechanism of Action of Electric and Electromagnetic Field Stimulation on Bone and Cartilage: New Findings and a Review of Earlier Work. J. Altern. Complement. Med..

[180] Kim I.S., Song J.K., Zhang Y.L., Lee T.H., Cho T.H., Song Y.M., Kim D.K., Kim S.J., Hwang S.J. (2006). Biphasic Electric Current Stimulates Proliferation and Induces VEGF Production in Osteoblasts. Biochim. Biophys. Acta Mol. Cell Res..

[181] Berchtold M.W., Villalobo A. (2014). The Many Faces of Calmodulin in Cell Proliferation, Programmed Cell Death, Autophagy, and Cancer. Biochim. Biophys. Acta Mol. Cell Res..

[182] Fassina L., Visai L., Benazzo F., Benedetti L., Calligaro A., Cusella De Angelis M.G., Farina A., Maliardi V., Magenes G. (2006). Effects of Electromagnetic Stimulation on Calcified Matrix Production by SAOS-2 Cells over a Polyurethane Porous Scaffold. Tissue Eng..

[183] Zhao M., Bai H., Wang E., Forrester J.V., McCaig C.D. (2004). Electrical Stimulation Directly Induces Pre-Angiogenic Responses in Vascular Endothelial Cells by Signaling through VEGF Receptors. J. Cell Sci..

[184] Nuccitelli R. (2003). A Role for Endogenous Electric Fields in Wound Healing. Curr. Top. Dev. Biol..

[185] Aaron R.K., McK Ciombor D., Keeping H., Wang S., Capuano A., Polk C. (1999). Power Frequency Fields Promote Cell Differentiation Coincident with an Increase in Transforming Growth Factor-b 1 Expression. Bioelectromagnetics.

[186] Aaron R.K., Wang S., Ciombor D.M.K. (2002). Upregulation of Basal TGFβ1 Levels by EMF Coincident with Chondrogenesis—Implications for Skeletal Repair and Tissue Engineering. J. Orthop. Res..

[187] Zhou L.L., Liu W., Wu Y.M., Sun W.L., Dörfer C.E., Fawzy El-Sayed K.M. (2020). Oral mesenchymal stem/progenitor cells: The immunomodulatory masters. Stem Cells Int..

[188] Liu N., Shi S., Deng M., Tang L., Zhang G., Liu N., Ding B., Liu W., Liu Y., Shi H. (2011). High levels of β-catenin signaling reduce osteogenic differentiation of stem cells in inflammatory microenvironments through inhibition of the noncanonical Wnt pathway. J. Bone Miner Res..

[189] Lim H.M., Nam M.H., Kim Y.M., Seo Y.K. (2021). Increasing Odontoblast-like Differentiation from Dental Pulp Stem Cells Through Increase of β-Catenin/p-GSK-3β Expression by Low-Frequency Electromagnetic Field. Biomedicines.

[190] Zhang F., Yan X., Wu M., Chen Y., Zhao H., Zhang C., Dang P., Wei L., Zhu F., Chen Y. (2024). Modulating Lineage Specification in Stem Cell Differentiation via Bioelectrical Stimulation Intensity Matching. Adv. Mater. Interfaces.

[191] Fathi E., Farahzadi R. (2017). Enhancement of osteogenic differentiation of rat adipose tissue-derived mesenchymal stem cells by zinc sulphate under electromagnetic field via the PKA, ERK1/2 and Wnt/β-catenin signaling pathways. PLoS ONE.

[192] Ferroni L., Gardin C., Dolkart O., Salai M., Barak S., Piattelli A., Amir-Barak H., Zavan B. (2018). Pulsed electromagnetic fields increase osteogenetic commitment of MSCs via the mTOR pathway in TNF-α mediated inflammatory conditions: An in-vitro study. Sci. Rep..

[193] Im A.L., Kim J., Lim K., Seonwoo H., Cho W., Choung P.H., Chung J.H. (2013). Effects of Micro-Electrical Stimulation on Regulation of Behavior of Electro-Active Stem Cells. J. Biosyst. Eng..

[194] Oliveira K.M.C., Leppik L., Keswani K., Rajeev S., Bhavsar M.B., Henrich D., Barker J.H. (2020). Electrical Stimulation Decreases Dental Pulp Stem Cell Osteo-/Odontogenic Differentiation. Biores. Open Access.

[195] Samiei M., Aghazadeh Z., Abdolahinia E.D., Vahdati A., Daneshvar S., Noghani A. (2020). The Effect of Electromagnetic Fields on Survival and Proliferation Rate of Dental Pulp Stem Cells. Acta Odontol. Scand..

[196] Rahimi S., Ahrabi M., Samiei M., Roshangar L., Ahrabi B., Hashemi B., Shahi S., Rahimi Darehchi N. (2023). The Effect of Low-Frequency Pulsed Electromagnetic Fields on the Differentiation of Permanent Dental Pulp Stem Cells into Odontoblasts. Iran Endod. J..

[197] Wang T., Wang P., Cao Z., Wang X., Wang D., Shen Y., Jing D., Luo E., Tang W. (2017). Effects of BMP9 and Pulsed Electromagnetic Fields on the Proliferation and Osteogenic Differentiation of Human Periodontal Ligament Stem Cells. Bioelectromagnetics.

[198] Costantini E., Marconi G.D., Fonticoli L., Aielli L., Trubiani O., Rajan T.S., Pizzicannella J., Reale M., Diomede F. (2022). Improved Osteogenic Differentiation by Extremely Low Electromagnetic Field Exposure: Possible Application for Bone Engineering. Histochem. Cell Biol..

[199] Lim K., Hexiu J., Kim J., Seonwoo H., Cho W.J., Choung P.H., Chung J.H. (2013). Effects of Electromagnetic Fields on Osteogenesis of Human Alveolar Bone-Derived Mesenchymal Stem Cells. Biomed. Res. Int..

[200] Cheng Y.C., Chen C.H., Kuo H.W., Yen T.L., Mao Y.Y., Hu W.W. (2019). Electrical Stimulation through Conductive Substrate to Enhance Osteo-Differentiation of Human Dental Pulp-Derived Stem Cells. Appl. Sci..

[201] Shi Z., Gao X., Ullah M.W., Li S., Wang Q., Yang G. (2016). Electroconductive Natural Polymer-Based Hydrogels. Biomaterials.

[202] Lu H., Zhang N., Ma M. (2019). Electroconductive Hydrogels for Biomedical Applications. Wiley Interdiscip. Rev. Nanomed. Nanobiotechnol..

[203] Rodrigues F., Rodrigues da Silva M., Silva F.S., Madeira S., Carvalho Ó. (2024). Electric Current Application on Dental Implant Biofilms: Review. J. Funct. Biomater..

[204] Jayasree A., Cartmell S., Ivanovski S., Gulati K. (2024). Electrically Stimulated Dental Implants Triggers Soft-Tissue Integration and Bactericidal Functions. Adv. Funct. Mater..

[205] Min Q., Gao Y., Wang Y. (2024). Bioelectricity in Dental Medicine: A Narrative Review. Biomed. Eng. Online.

[206] Titushkin I., Cho M. (2009). Regulation of Cell Cytoskeleton and Membrane Mechanics by Electric Field: Role of Linker Proteins. Biophys. J..

[207] Durand D.M. (2014). Electrical Stimulation of Excitable Tissue. Biomedical Engineering Fundamentals.

[208] Zhou X., Li G., Wu D., Liang H., Zhang W., Zeng L., Zhu Q., Lai P., Wen Z., Yang C. (2023). Recent Advances of Cellular Stimulation with Triboelectric Nanogenerators. Exploration.

[209] Liu X., Wan X., Sui B., Hu Q., Liu Z., Ding T., Zhao J., Chen Y., Wang Z.L., Li L. (2024). Piezoelectric Hydrogel for Treatment of Periodontitis through Bioenergetic Activation. Bioact. Mater..

[210] Roldan L., Montoya C., Solanki V., Cai K.Q., Yang M., Correa S., Orrego S. (2023). A Novel Injectable Piezoelectric Hydrogel for Periodontal Disease Treatment. ACS Appl. Mater. Interfaces.

[211] Hashimoto H. (1990). Effect of Micro-Pulsed Electricity on Experimental Tooth Movement. Orthod. Waves.

[212] Wang Y., Zhou J., Chen K., Yu X., Dong Z., Liu Y., Meng X. (2024). Electrical Stimulation Induced Pre-Vascularization of Engineered Dental Pulp Tissue. Regen. Ther..

[213] Spadari G.S., Zaniboni E., Vedovello S.A.S., Santamaria M.P., do Amaral M.E.C., dos Santos G.M.T., Esquisatto M.A.M., Mendonca F.A.S., Santamaria-Jr M. (2017). Electrical Stimulation Enhances Tissue Reorganization during Orthodontic Tooth Movement in Rats. Clin. Oral Investig..

